# The Bayesian Mutation Sampler Explains Distributions of Causal Judgments

**DOI:** 10.1162/opmi_a_00080

**Published:** 2023-06-15

**Authors:** Ivar R. Kolvoort, Nina Temme, Leendert van Maanen

**Affiliations:** Department of Psychology, University of Amsterdam, Amsterdam, The Netherlands; Institute for Logic, Language, and Computation, University of Amsterdam, Amsterdam, The Netherlands; Department of Experimental Psychology, Utrecht University, Utrecht, The Netherlands

**Keywords:** causal reasoning, causal judgments, priors, sampling, response distributions, conservatism

## Abstract

One consistent finding in the causal reasoning literature is that causal judgments are rather variable. In particular, distributions of probabilistic causal judgments tend not to be normal and are often not centered on the normative response. As an explanation for these response distributions, we propose that people engage in ‘mutation sampling’ when confronted with a causal query and integrate this information with prior information about that query. The Mutation Sampler model (Davis & Rehder, 2020) posits that we approximate probabilities using a sampling process, explaining the average responses of participants on a wide variety of tasks. Careful analysis, however, shows that its predicted response distributions do not match empirical distributions. We develop the Bayesian Mutation Sampler (BMS) which extends the original model by incorporating the use of generic prior distributions. We fit the BMS to experimental data and find that, in addition to average responses, the BMS explains multiple distributional phenomena including the moderate conservatism of the bulk of responses, the lack of extreme responses, and spikes of responses at 50%.

## INTRODUCTION

Causal reasoning is a core facet of human cognition. Dealing with causal relationships in the world and using them to our advantage is a crucial part of our abilities. Causal cognition ties into most (if not all) judgments and decisions (e.g., Hagmayer & Osman, 2012; Rottman & Hastie, 2014). Most of what we do and think is at least partly based on the perception of and reasoning about causes and effects in the world. This makes it an important aim in cognitive science to understand how we think and reason about causes and effects.

The current work addresses probabilistic causal reasoning. An example of this is when someone tries to judge the probability that they will be late for work after hearing on the radio that there has been a traffic accident nearby. To make such a judgment one needs to use their knowledge of a causal system. In this case such knowledge could be represented as *X* → *Y* → *Z*, where *X* stands for a traffic accident, *Y* for a traffic jam, and *Z* for being late for work. In a typical probabilistic causal reasoning experiment people are first taught about a particular causal system (for example *X* → *Y* → *Z*), after which they are asked to make certain inferences, i.e., compute certain (conditional) probabilities. An example of such an inference is “what is the probability of *Z* (being late for work) given that *X* (a traffic accident) happened?”. These experiments provide a window into how participants make use of causal information to come to a specific judgment. The current work will focus on using cognitive modelling to understand how people make such judgments using their causal knowledge.

Over the last decades so-called causal Bayesian networks^1^ (CBNs; Pearl, 2009; Spirtes et al., 2000) have achieved remarkable success in modeling human behavior across a variety of tasks related to causal learning, categorization, reasoning and inference (e.g., Ali et al., 2011; Bramley et al., 2015; Cheng, 1997; Coenen et al., 2015; Fernbach & Erb, 2013; Griffiths & Tenenbaum, 2005, 2009; Hagmayer, 2016; Hayes et al., 2014; Holyoak et al., 2010; Krynski & Tenenbaum, 2007; Lee & Holyoak, 2008; Lu et al., 2008; Meder et al., 2014; Rehder, 2014; Rehder & Burnett, 2005; Shafto et al., 2008; Steyvers et al., 2003; Waldmann & Hagmayer, 2006). These models provide a concise representation of causal systems and a particular formal logic that specifies how one can learn, draw inferences, and update causal knowledge based on interventions in the system.

CBNs, as models of human cognition, are often understood as explanations on the computational level (Marr, 1982). That is, they provide an account of what problem needs to be solved, but not how to solve them. This is because formal computations with CBN models tend to be computationally expensive and so are thought not be feasible as a way for us humans to solve problems. Instead of directly doing these Bayesian computations, recent work in the cognitive sciences has argued that people use sampling to solve such computationally intensive problems in a variety of domains (Bonawitz et al., 2014; Dasgupta et al., 2017; Hertwig & Pleskac, 2010; Lieder et al., 2012; Vul et al., 2014; Zhu et al., 2020). This *sampling approach to cognition* proposes that we solve problems by way of first drawing samples, either from memory or an internal generative model, and then generating judgments based on the information in these samples. In this way we can reason about probabilities without the need to explicitly represent probabilities. Often the sampling approach is modelled using Markov chain Monte Carlo (MCMC) processes (see Dasgupta et al., 2017). Davis and Rehder (2020) applied this sampling approach to the domain of causal cognition, developing a model that samples over possible states of a CBN to make causal judgments. This so-called Mutation Sampler model (MS) provides an algorithmic level explanation (Marr, 1982) as it describes *how* humans reason causally. It proposes a sampling mechanism for how we generate causal judgments and has been successful in explaining average responses on a variety of tasks (Davis & Rehder, 2020).

However, while accounts of average responses abound, the common observation of substantial variability in causal judgments has received less attention and is often left unexplained (Davis & Rehder, 2020; Kolvoort et al., 2021, 2022; Rehder, 2014, 2018; Rottman & Hastie, 2016). This is an unfortunate gap in the literature, as variability in behavior can be informative of the cognitive mechanisms involved and so can help constrain the development of theories (e.g., as has been done in the domain of decision-making; Ratcliff, 1978). In this paper, we will analyze the distributional predictions of the MS and ultimately extend it with the incorporation of priors to provide an explanation of some of the observed variability in causal judgments.

### Sampling Theory and the Mutation Sampler

The MS is a sampling model of causal reasoning that accounts for many observed behavioral phenomena including deviations from the normative CBN model (Davis & Rehder, 2017, 2020; Rehder & Davis, 2021). Before discussing the model in more detail, it is important to note that the original authors argue for four psychological principles to govern causal reasoning and that the MS is but one formalization of these principles. We will refer to these principles in this section, for a more detailed discussion on these psychological claims and the exact formalization of the MS we refer to the original paper (Davis & Rehder, 2020).

**Figure 1.**
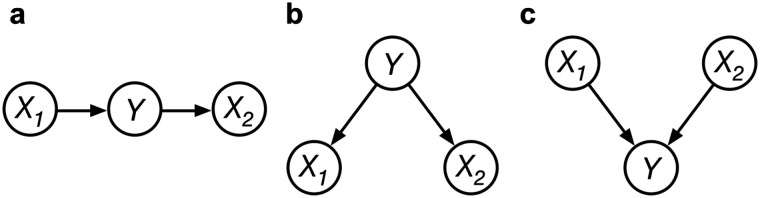
**Three-variable causal networks.** The circles represent causal variables, the arrows represent causal relationships. Throughout this manuscript we will use *Y* to refer to the middle variable, and *X*_*i*_ to refer to a terminal variable. (A) Chain structure. (B) Common cause structure. (C) Common effect structure.

The MS proposes that people engage in sampling over states of a causal network to make inferences and as such describes the process by which people generate causal judgments. This proposal is built on the psychological claim that people reason about concrete cases and not about abstract probabilities (Principle 1). The concrete cases here are causal networks instantiated with particular values, i.e., they are particular casual network states (see Figure 1 for three-variable causal networks). These concrete cases or causal network states are obtained from memory or through simulations using an internal generative model. Subsequently these samples are used to compute inferences based on the relative frequencies of certain events in the chain of generated samples.

The MS generates a sequence of samples of a causal system using a Metropolis-Hastings MCMC sampling algorithm (Davis & Rehder, 2020). The sampler starts in one of two prototypical states, with equal probability. The prototypical states are states in which the causal variables are either all present or all absent (i.e., for 3-variable causal networks, [*X*_1_ = 1, *Y* = 1, *X*_2_ = 1] and [*X*_1_ = 0, *Y* = 0, *X*_2_ = 0]). Next, a new state is proposed from the states that differ only in value of a single variable from the current state, i.e., the current state is ‘mutated’. This implements the idea that people tend to only make small adjustments to the cases they are considering currently (Principle 2). Whether the next sample is this proposal state or is the current state is determined by the transition probability. The transition probability is defined by the relative likelihood of the proposal and current state (Hastings, 1970). If the proposal state is more probable than the current state, the next sample will be the proposed state with probability 1. If the proposal state is less probable than the current state, the probability of the next sample being the proposed state is determined by the ratio of the joint probabilities of the proposed and current state. Due to the transitions being defined by probabilities this is a stochastic process.

The chain of samples generated by this scheme converges to reflect the actual normative joint distribution when the number of samples becomes sufficiently large. This means that judgments based on a large number of samples approximate the true probabilities. However, people do not respond normatively and nor does the MS. Two factors contribute to the non-normativity of judgments based on the mutation sampling process: (1) the starting point of the process is biased (to prototypical states) and (2) the number of samples (or ‘chain length’) is limited.

Both these factors, limited sampling and biased starting points, lead to a probability distribution that overestimates the likelihood of states where more variables have the same value. That is, it is biased towards prototypical states (see Figure 3 in Davis & Rehder, 2020). Since a chain of samples always starts at a prototypical state and has limited opportunity to reach states that are very different from these prototypical states (due to limited number of samples), the predicted probability distribution places more probability density on states based on their closeness to the prototypical states. This effect is stronger when the number of samples taken is small.

Davis and Rehder (2020) provide psychological justifications for these aspects of their model that lead to non-normative responding. With regard to the biased starting points, Davis and Rehder suggest that “prototypes readily come to mind as plausible states at which to start sampling because, if one ignores the details of the causal graph such as the strength, direction, or functional form of the causal relations, they are likely to be viewed as having relatively high joint probability” (Principle 3; Davis & Rehder, 2020, pp. 6). Assuming generative causal links, this is the case because a prototypical state is always consistent with the causal relationships in that there are no cases in which effects are absent while their causes are present and vice versa. Moreover, these prototype states are generally high probability states and so are good starting points for convergence. Taken together, when we start thinking about a causal system we start in a simple state, that is likely to be remembered or generated as it occurs often and is consistent with all causal relationships in the network.

The second aspect of the MS that leads to non-normative responding is that the chains of samples are of limited length (Principle 4). Other work on sampling approaches to cognition has shown that using only a limited number of samples can be rational when taking into account costs associated with taking more samples (see Dasgupta et al., 2017; Hertwig & Pleskac, 2010; Vul et al., 2014) As such the MS can be viewed as part of the new resource-rational analysis modeling paradigm in which the rational use of limited resources is a guiding principle (Lieder & Griffiths, 2020).

Davis and Rehder (Davis & Rehder, 2020; Rehder & Davis, 2021) fitted the MS to data from a variety of experiments concerning causal reasoning, categorization, and intervention. They found that the model fits better to participant responses than standard CBNs. Moreover, the model is able to account for multiple systematic reasoning errors, i.e., deviations from the normative CBN model, by virtue of the limited sampling and biased starting point mechanisms. For instance, the distorted joint distribution that the MS produces is able to account for Markov violations and failures to explain away, two hallmark behavioral phenomena in causal reasoning (Kolvoort et al., 2021, 2022; Park & Sloman, 2013; Rehder, 2018; Rehder & Burnett, 2005; Rottman & Hastie, 2014, 2016). In addition to using existing data, Davis and Rehder (2020) ran an experiment that presented participants with causal graphs and asked them to generate data that they thought would be consistent with the causal structure. The data participants generated matched the distorted joint distributions produced by the MS. Taken together, the tested predictions of the MS seem to be in very good accord with experimental data.

However, not all aspects of the of predictions have been scrutinized. As the MS posits that the transitions from one state to another in the generation of a chain of samples are stochastic, it predicts not just a mean response (like the CBN estimate), but a full distribution of responses to an inference. This is in line with the MS being a process-model, since it models the process by which people generate causal judgments it should also produce the variability in responses seen on a variety of tasks (Davis & Rehder, 2020). However, following the literature on causal cognition at large, these distributional predictions have not yet received proper attention. The aim of the current paper is to assess these distributional predictions and use the distributional phenomena in empirical data to guide further development of the MS.

The rest of this paper is structured in two main parts. In the first part we analyze the distributions predicted by the MS and find that it cannot explain certain distributional phenomena observed in causal reasoning tasks. In addition, we provide theoretical arguments against the (resource-)rationality of the model, leading us to extend the model using priors in the second part. In the second part we introduce the Bayesian Mutation Sampler and test its distributional predictions. We conclude with a general discussion concerning both the theoretical and empirical advances made in this paper.

## ANALYZING THE MS AND DISTRIBUTIONAL PHENOMENA

The nature of the MS as a process-model makes it a useful tool to assess distributional phenomena in addition to the mean phenomena that have been extensively studied (see Kolvoort et al., 2021). It has been observed multiple times that causal judgments vary substantially (Davis & Rehder, 2020; Kolvoort et al., 2021; Rehder, 2014, 2018; Rottman & Hastie, 2016). The MS has not been used to study distributional properties of responses. The authors did present a figure indicating a qualitative similarity between the variability of responses in Experiments 1A and 1B by Rottman and Hastie (2016) and the variability of the predicted responses by the MS with a mean chain length of 36 (see Figure 9 in Davis & Rehder, 2020). However, this value for the chain length is far from the average best fitting parameter (which was 12.7) that the authors found for a range of causal reasoning experiments (Davis & Rehder, 2020). Therefore, many questions remain regarding the predicted distributions of the MS. Here, we aim to assess these predictions under a range of different chain lengths.

**Figure 2.**
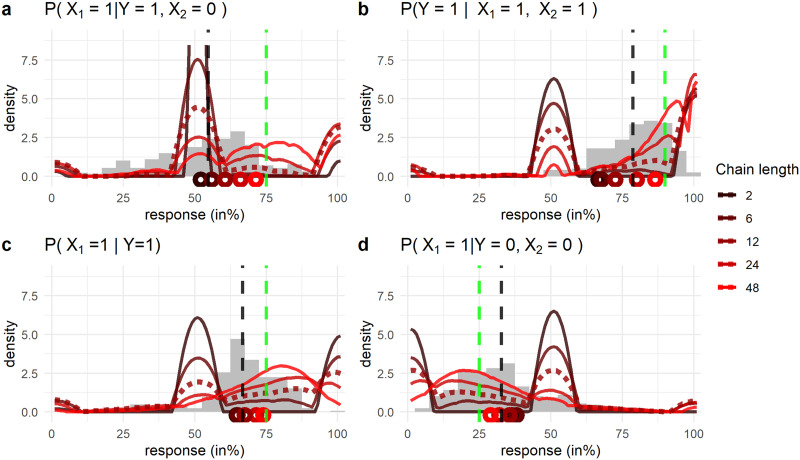
**Predictions of the Mutation sampler and data from Kolvoort et al. (2022) on four different inferences.** (A) *P*(*X*_1_ = 1|*Y* = 1, *X*_2_ = 0); (B) *P*(*Y* = 1|*X*_1_ = 1, *X*_2_ = 1); (C) *P*(*X*_1_ = 1|*Y* = 1); (D) *P*(*X*_1_ = 1|*Y* = 0, *X*_2_ = 0). Plots A–D: The inferences were about a common cause structure where *X*_1_ and *X*_2_ refer to the two effects, and *Y* refers to the common cause. A state of 1 (e.g., *Y* = 1) indicated a variable was present, a state of 0 indicated the variable was absent. Grey histograms are the participant responses. The red lines indicate predictions of the MS with different chain lengths. The dashed red line are the predictions with chain length of 12, close to the mean found by Davis and Rehder (2020). The predicted response distributions were generated by simulating 10,000 responses with the MS and smoothing the result using kernel density estimation. Vertical dashed black lines indicate mean participant response. Dashed green lines indicate the normative answer. The colored circles on the *x* axis indicate mean predictions.

To this end we simulated responses using the MS with multiple chain lengths using the same causal parameters^2^ as in Experiment 1A by Rottman and Hastie. We chose these parameters since the MS was fitted to that experiment originally and they are intended to theoretically neutral (Rottman & Hastie, 2016). Figure 2 present the results of the simulations together with empirical data from a recent causal reasoning experiment (Kolvoort et al., 2022). These data are causal probabilistic judgments, where participants had to judge the probability of a causal variable being present conditional on information about other causal variables in the network (e.g., *P*(*X*_1_ = 1|*Y* = 1)). The experiment is described in more detail in a later section, for now it suffices to note that it used similar methods, including the exact same causal parameters, as Experiment 1A by Rottman and Hastie (2016).

### Mutation Sampler Predicts Extreme Responses and No ‘Moderate’ Conservatism

Let us take the predictions of the MS with chain length 12 (dashed red lines in Figure 2) as a starting point for our discussion, since 12 is close to the mean chain length found for causal reasoning tasks (Davis & Rehder, 2020). What immediately stands out in Figure 2 is that the MS with a chain length of 12 predicts three peaks of responses for each inference at 0%, 50% and 100%. Spikes of responses at 50% have been reported in the literature on causal judgments (Kolvoort et al., 2021; Rottman & Hastie, 2016). To the contrary, the peaks at 0% and 100% seem not to correspond to empirical data. In fact, it is known that participants behave conservatively and tend to avoid the extremes of the scale (Kolvoort et al., 2021, 2022; Rottman & Hastie, 2016). This makes the predictions at 0% and 100% rather surprising.

To understand these predictions we have to take a closer look at the mechanisms causing these peaks. Firstly, the peak at 50%. These peaks are due to the MS defaulting to a 50% response for conditional probability queries when the causal network states required for the calculation are not reached at any point by the stochastic sampling process. Throughout this manuscript we will use *Y* to refer to middle variable, and *X*_*i*_ to refer to a terminal variable (see Figure 1). Let us say the required inference is *P*(*X*_1_ = 1|*Y* = 1, *X*_2_ = 0) as in Figure 2A. In this case the sampler needs to visit the states [*X*_1_ = 1, *Y* = 1, *X*_2_ = 0] and [*X*_1_ = 0, *Y* = 1, *X*_2_ = 0] to compute the inference based on the relative frequency of these states in the chain. This computation is done using the (Kolmogorov) definition of conditional probability:

$$P\left(X_{1}=1│Y=1,X_{2}=0\right)=\frac{P\left(X_{1}=1,Y=1,X_{2}=0\right)}{P\left(X_{1}=1,Y=1,X_{2}=0\right)+P\left(X_{1}=0,Y=1,X_{2}=0\right)}$$

From which we can get an estimate for the conditional probability based on sample frequencies:

$$\hat{P}\left(X_{1}=1│Y=1,X_{2}=0\right)=\frac{N\left(X_{1}=1,Y=1,X_{2}=0\right)}{N\left(X_{1}=1,Y=1,X_{2}=0\right)+N\left(X_{1}=0,Y=1,X_{2}=0\right)}$$ (1)

where *N* stands for the number of samples of that causal state. Now if the required states on the right-hand side of Equation 1 are not visited their frequencies (or probabilities) are zero. In this case Equation 1 would reduce to $\frac{0}{0}$ which cannot be computed and instead the MS defaults to 50%^3^.

The predicted peaks at 0% and 100% come about similarly as the peak at 50%, however in this case only one of the two required states is not visited. Let us again consider the inference *P*(*X*_1_ = 1|*Y* = 1, *X*_2_ = 0), which requires the state A: [*X*_1_ = 1, *Y* = 1, *X*_2_ = 0] and B: [*X*_1_ = 0, *Y* = 1, *X*_2_ = 0] to be visited by the sampler. In the case where state A is not visited, Equation 1 simplifies to $\frac{0}{0+P\left(X_{1}=0,Y=1,X_{2}=0\right)}$ and we get a response at 0%. When state B is not visited by the sampler, Equation 1 simplifies to $\frac{P\left(X_{1}=1,Y=1,X_{2}=0\right)}{P\left(X_{1}=1,Y=1,X_{2}=0\right)+0}$ and we get a predicted response at 100%.

To gain insight in how often the MS generates ‘default’ responses at 0%, 50%, or 100% we can estimate how often a particular network state is expected to be visited. We do this by simulating 10,000 chains of samples. Let us again regard *P*(*X*_1_ = 1|*Y* = 1, *X*_2_ = 0) (Figure 2A), which requires visits to the states A: [*X*_1_ = 1, *Y* = 1, *X*_2_ = 0] and B: [*X*_1_ = 0, *Y* = 1, *X*_2_ = 0] to be computed (see Equation 1). We find that with a chain length of 12, the proportion of trials on which state A is not visited by the sampler is 0.49, the proportion where state B is not visited is 0.72, and the proportion of trials where neither is visited is 0.39. As a direct result, we see more responses predicted at 100% than at 0% in Figure 2A, as it is more likely for state B to not be visited than state A. Only in 18% of the trials does the sampler actually reach both state A and B, meaning that in only 18% of the judgments a probability estimate is computed by comparing the nonzero frequencies of states A and B in the chain of samples. We will refer to these as ‘computed’ responses to contrast them from what we will refer to as ‘default’ responses at 0%, 50%, or 100% which occur when at least one state (A or B) was not visited by the sampler. The other 82% of the time the MS predicts such default responses at 0%, 50%, or 100%, which is why we observe large peaks in the dashed line at 0%, 50%, and 100% in Figure 2.

Let us now look at the effect of different chain lengths. As the predicted peaks at 0%, 50% and 100% by the MS are all due to certain network states not being visited by the sampling process, the number of samples drawn, i.e., the chain length, determines the size of these predicted peaks. Fewer samples drawn increases the probability that certain states are not visited and so larger peaks are predicted^4^. This effect of the chain length on predicted response distributions can be seen from the multiple red lines in Figure 2: Longer chain lengths, indicated by brighter red lines, have smaller peaks and provide a mean estimate that is closer to the normative answer. To assess the effect of chain length on the amount of default and computed responses we again simulated 10,000 runs with the MS, this time using chain lengths ranging from 2 to 48 (Table 1).

**Table 1.** Predicted responses of the Mutation Sampler for the inference *P*(*X*_1_ = 1|*Y* = 1, *X*_2_ = 0).

**Chain length**	**Probability of response**			
	**Computed**	**0%**	**50%**	**100%**
2	0.0	0.0177	0.927	0.0554
6	0.0605	0.0599	0.667	0.213
12	0.182	0.0936	0.392	0.332
24	0.418	0.0870	0.146	0.349
48	0.729	0.0309	0.0228	0.218

Probabilities are calculated by running the Mutation Sampler 10,000 times using the causal parameters of the common cause network in Experiment 1A in Rottman and Hastie (2016). ‘Computed’ refers to responses that are computed by comparing the relative frequency of network states. We contrast these with ‘default’ responses at 0%, 50%, or 100%.

As mentioned before, smaller chain lengths make it less likely that the required causal network states to compute an inference are visited by the sampler. With a chain length of two, the darkest red lines in Figure 2, the two states required to compute the inference are never both visited and the MS predicts responses to be exclusively at 0%, 50%, or 100%. From Table 1 we can indeed see that the *P*(*X*_1_ = 1|*Y* = 1, *X*_2_ = 0) inference was never computed in 10,000 runs with a chain length of 2. For larger chain lengths these peaks are present as well albeit with a smaller magnitude. Even with a chain length of 48, which is at the high end of the range of chain lengths found previously (Davis & Rehder, 2020), the MS still predicts a substantial proportion of extreme responses at 0 and 100%. For instance, for the inference *P*(*X*_1_ = 1│*Y* = 1, *X*_2_ = 0) the MS predicts responses at 100% for all chain lengths simulated (Figure 2A and Table 1). Even with a chain length of 48 the MS still predicts 22% of responses to be an extreme response of 100%. For each chain length above 2 the probability of an extreme response is at least 24% (Table 1). These predictions of the MS are not borne out, multiple studies have found participants to avoid the extremes of the scale in causal reasoning studies (e.g., Kolvoort et al., 2021, 2022; Rottman & Hastie, 2016).

Figure 2 illustrates another aspect of the MS predictions that do not match the empirical data. We know that when the chain lengths are larger the predicted response peaks at 0%, 50%, and 100% decrease while the proportion of computed responses increases (Table 1). Regarding these computed responses, when the number of samples, i.e., the chain length, tends to infinity the predicted responses will tend towards the normative CBN point prediction. Hence, the mean prediction will get closer to the normative response with increasing chain lengths. One can see this happening from the red circles on the *x* axis indicating the mean predicted response in Figure 2. This indicates that there is a tradeoff in the predicted distributions of the MS between the peaks at 0%, 50%, 100% and a peak of computed responses that gets closer to the normative response when chain lengths increase.

Based on this tradeoff the MS can predict mean conservatism by varying chain lengths. With very low chain lengths, the mean response is close to 50% as most responses will be default responses at 50%. At large chain lengths the mean response will approach the normative response. With more moderate chain lengths the mean response will lie in between 50% and the normative response (see circles on *x* axis in Figure 2). This observation is consistent with the literature, as mean participant responses tend to be conservative and lie between 50% and the normative answer. However, it is not just the mean response that is between 50% and the normative answer. Typically, the bulk of responses tends to lie between 50% and the normative answer (Kolvoort et al., 2022; Rottman & Hastie, 2016). This however is inconsistent with the MS prediction, as the MS only predicts mean conservatism by trading off default responses (mainly at 50%) for computed responses (near the normative response). It is not able to predict the bulk of moderately^5^ conservatism responses in between 50% and the normative answer that have been found in experiments (Kolvoort et al., 2021, 2022; Rottman & Hastie, 2016). Relatedly, the model can predict some variability in responses that is similar to the empirical distributions (see predicted distributions with chain lengths 24 and 48 in Figure 2), however when it does so the mean predicted response tends to be off and there are still peaks that are not present in participant’s responses. The mechanics of the MS lead to distributions that cannot mimic empirical responses in terms of certain distributional phenomena. To serve as an complete explanation of the cognitive process, the MS should be able to predict distributional behavioral phenomena in addition to mean phenomena.

This analysis of response distributions brought to light two important aspects of the data that the MS currently does not account for. The first is that it predicts extreme responses with a wide range of chain lengths. These responses are not observed in experiments, where people shy away from the extreme ends of the scale (Kolvoort et al., 2021, 2022; Rottman & Hastie, 2016). The second issue has to do with participant’s conservatism: the bulk of responses is between 50% and the normative answer (Kolvoort et al., 2022; Rottman & Hastie, 2016). The mutation sampler can predict mean conservatism, but seemingly only by balancing the size of the peaks at 0%, 50%, and 100% with computed responses. It does not predict the bulk of responses to be in between the normative answer and 50%.

### Forming Judgments Based on Samples

To better understand how we could resolve the issues of the MS we identified above we consider the process of forming judgments based on samples. The most straightforward manner in which people can form probability judgments based on a set of samples is by calculating the relative frequency of an event occurring in the samples and taking this as an estimate of a probability of the occurrence of that event (Zhu et al., 2020). To illustrate this, imagine a scenario in which someone repeatedly throws tennis balls at beer bottles causing some to break. To estimate the probability that the bottle breaks with the next throw, we can compute the frequency of the bottle breaking in samples where a tennis ball is thrown:

$$\text{Relative}\;\text{frequency}=\frac{N_{\text{breaks}}}{N_{\text{breaks}}+N_{\text{does}\;\text{not}\;\text{break}}}=\frac{N_{\text{breaks}}}{N_{\text{throws}}}$$

So, for instance, if bottles break 12 times in 20 throws, the relative frequency of the bottle breaking is 12/20 = 0.6. Then, someone using the relative frequency approach would judge the probability of a bottle breaking as 0.6. This entails that a judgment is completely based on the incoming information and the judgment would approach the true probability when the number of samples tends to infinity. The MS uses this relative frequency method, computing judgments directly from the relative frequency of samples.

Problems with the relative frequency approach arise when we look at what happens when the number of samples is limited or small. According to the relative frequency method one could judge the probability of an event that is only witnessed once to be occurring 100% of the time. That is, if one observes a bottle to break after only one throw, one would judge the probability of a ball causing a bottle to break to be 100%. The reverse is also the case, the relative frequency method would lead one to judge anything that has not been directly observed yet to occur with a 0% probability. These extreme judgments are psychologically implausible. A more psychologically plausible model is to include prior information. The use of prior information can stop us from making extreme responses when we have little information to go by.

Besides preventing extreme judgments there is a more normative argument for the use of priors. The use of priors matches our decision-making in that it allows for gradual adjustments in the face of consistent evidence. When more and more tennis balls consistently break bottles, a judgment of 100% becomes more reasonable. This illustrates that people are sensitive to the amount of information obtained. When incorporating prior information, the amount of evidence presented (here the number of samples of throws, or ‘likelihood’ in Bayesian terms) does directly impact one’s judgment because we can weigh it relative to our prior information (e.g., our estimate gradually moves to 100% after seeing bottles break consistently). In contrast, the relative frequency approach would be insensitive to the amount of information learned from sampling.

Based on the previous theoretical arguments and the problems the MS has with predicting empirical response distributions, we propose the Bayesian Mutation Sampler (BMS) as an account of how people make causal judgments. The BMS is a process-model of causal reasoning combining mutation sampling (Davis & Rehder, 2020) with a generic Bayesian approach using priors to make probability judgments from samples (Zhu et al., 2020). We expect that the incorporation of priors will help in explaining the distributional behavioral phenomena discussed in previous sections.

In the next section, we will give a detailed overview of the BMS and subsequently will test whether it is an improvement over the MS, particularly in terms of the prediction of distributional properties, by fitting both models to experimental causal reasoning data.

## THE BAYESIAN MUTATION SAMPLER

The BMS posits that when making causal probabilistic judgments people engage in sampling by way of mutation sampling (Davis & Rehder, 2020). This includes the principles of limited sampling and biased starting points, which bias judgments away from the normative CBN response. However, instead of using the relative frequency method to form judgments based on samples (as in the MS), the BMS incorporates prior information.

The type of prior information that people use for judgments and decision-making varies. Many causal reasoning studies attempt to exclude the use of prior information regarding causal model parameters (e.g., Kolvoort et al., 2022; Rehder, 2014; Rottman & Hastie, 2016). However, even if researchers are successful in stopping participants from using prior information concerning causal parameters, it is likely that people still inherit priors relevant to the experimental task from similar everyday activities or in some way or another have expectations concerning the experimental task (see Hemmer et al., 2015; Marchant et al., 2021; Sanborn et al., 2021; Tauber et al., 2017; Welsh & Navarro, 2012). When specific task-related information is not present people can still use priors that reflect a lack of information.

The BMS posits that reasoners use a generic prior that encodes what they think to be likely answers to a causal probabilistic query before sampling. This prior gets updated based on the information in the samples. In Bayesian terms, the prior is updated using the information in the samples (the likelihood) to produce a posterior distribution. Subsequently probability judgments are based on this posterior distribution. Following Zhu et al. (2020), we take it that people respond using the expected value of this distribution (see also Jazayeri & Shadlen, 2010).

### Incorporating the Symmetric Beta Prior

Following Zhu et al. (2020) we use symmetric Beta distributions as priors in the BMS, as they can reflect a lack of information in various ways and because they can be naturally incorporated into sample frequencies to form judgments.

**Figure 3.**
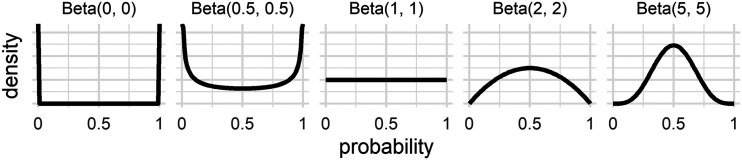
**Symmetric Beta(*β*, *β*) distributions, using *β* = 0, 0.5, 1, 2, and 5.** Symmetric Beta distributions will be used as prior distributions in the BMS.

Figure 3 plots symmetric Beta(*β*, *β*) distributions with values for *β* as the shape parameters. The Beta(1, 1) distribution is the uniform distribution, assigning equal probability mass to each probability p (from 0 to 1). For *β* > 1 the beta distributions assign more probability mass to the center of the scale, i.e., probabilities around .5. *β* < 1 shows the opposite pattern, where more probability is assigned to the extreme ends of the scale. In this way using the symmetric Beta distributions allows the BMS to account for various levels of conservatism.

For all *β* > 0 the incorporation of the prior moves a response closer to 50% than when just using the relative frequency method. The only symmetric Beta distribution that would not introduce conservatism in this sense is the Beta(0, 0) distribution where all the probability mass is at the extremes of the range, at 0 and 1. Using the Beta(0, 0) distribution is equivalent to using the relative frequency method of forming judgments from samples. This entails that the BMS with *β* set to 0 is equivalent to the standard MS and so the BMS is a generalization of the MS.

By using the Beta(*β*, *β*) distribution as a prior, the expected value of the posterior distribution can be determined without computing the posterior distribution itself. We can compute the expected value directly by adding *β* as ‘pseudo-observations’ to Equation 1 as in Equation 2 (for the derivation of Equation 2 we refer to Appendix A in Zhu et al., 2020)^6^.

$${\hat{P}}_{BMS}\left(X_{1}=1│Y=y,X_{2}=x\right)=\frac{N\left(X_{1}=1,Y=y,X_{2}=x\right)+\beta }{N\left(X_{1}=1,Y=x,X_{2}=y\right)+N\left(X_{1}=0,Y=x,X_{2}=x\right)+2\beta }$$ (2)

In Equation 2 $\hat{P}$_*BMS*_ refers to the estimate of the probability of an event predicted by the BMS, N stands for the number of samples (in a chain of generated samples), and *X*_*i*_, *Y* refer to causal variables and *x*, *y* refer to their respective states. The *β* refers to the Beta(*β*, *β*) prior used, where both shape parameters of the Beta distribution are equal to *β*.

### Testing the BMS

In order to validate whether the BMS provides a better explanation of response distributions than the MS, while still being able to predict mean responses as accurately as the MS, we fitted both models to data from a recent causal reasoning experiment (Kolvoort et al., 2022).

**Figure 4.**
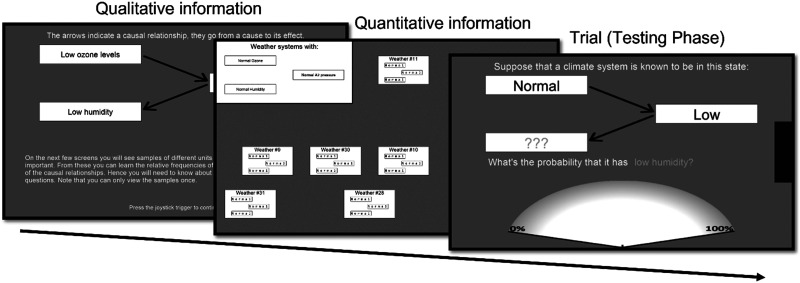
**Overview of experiment in Kolvoort et al. (2022) with screenshots.** First participants are taught about a particular causal system, receiving both qualitative (screen 1) and quantitative (screen 2) information on the causal variables, causal relationships, and causal strengths. Next, participants are asked to respond to (conditional) probability queries (screen 3).

Here we provide a brief description of the experimental data, for a more detailed discussion we refer to the original paper (Kolvoort et al., 2022). The experiment consisted of three experimental domains, each comprising a learning phase and a testing phase. In the learning phase participants learned a specific causal structure, about which they were asked to make inferences in the testing phase (Figure 4).

In the learning phase participants were provided with information about a causal system with three binary variables. They were given qualitative information concerning the variables and causal relations, as well as quantitative information using the experience sampling method with data sheets (Rehder & Waldmann, 2017) which involves participants viewing samples of data that manifest the statistical relations implied by the causal model. Each of the experimental domains used a different causal structure, which was either a chain, common cause, or common effect structure. The network parametrization of the structures was taken from Rottman and Hastie (2016, Experiment 1A), which was also used to fit the original MS to (Davis & Rehder, 2020).

In the testing phase participants responded to (conditional) probabilistic queries regarding the causal systems. Each of the 3 testing phases consisted of three blocks with different levels of time pressure implemented using response deadlines of 3, 9 and 20 seconds (of which the last one was intended to give participants ample time to respond). Each of these blocks consisted of 27 trials each consisting of a different inference. These inferences were of the form ‘Variable A has value *x*, variable B has value *y*. What is the probability that variable C has value *z*?’. Each of the three variables could have three states, one of the two binary values or unknown, leading to 3^3^ = 27 different inferences. All participants completed 27 trials per domain and deadline condition, for a total of 27 × 3 × 3 = 243 trials. Participants responded on a scale from 0% to 100%.

Out of the 43 participants in the dataset, 17 did the study online. The only noteworthy difference between the online and offline study was the response modality; participants in the lab indicated a percentage by moving a joystick while online participants responded by moving their cursor using a mouse or trackpad.

We will fit the BMS and MS to each participant and condition (response deadline × causal structure) separately, this results in 43 × 3 × 3 = 387 sets of fitted parameters. In this way each set of parameters is fitted to 27 responses on 27 different inferences of a participant.

We did not identify a closed-form likelihood function for the BMS. Moreover, the parameters of the models consist of a combination of continuous (beta) and discrete (chain length) parameters. These considerations suggest a discontinuous or at least complex parameter landscape. A parameter recovery study (Appendix A) supported this suspicion, but revealed that using a parameter grid search (cf. Maaß et al., 2021; Mestdagh et al., 2019) resulted in correlations between true and estimated parameters consistently above .75 (see Method 2 Coarse grid in Appendix A), which is generally seen as good or excellent recovery (e.g., Anders et al., 2016; van Maanen et al., 2021; van Ravenzwaaij & Oberauer, 2009; White et al., 2018). Moreover, the parameter recovery study provided assurance regarding the identifiability of the BMS (van Maanen & Miletić, 2021).

To fit the models using a grid search, we first simulate responses using the models with a range of realistic parameters (see below). These simulated responses were then saved in a grid. Each cell of this grid represents the predictions of the model under a particular set of parameters. To compute how closely the simulated responses match empirical responses we use the Probability Density Approximation method (PDA; Holmes, 2015; Turner & Sederberg, 2014) on each grid cell. PDA computes a ‘synthetic’ likelihood through kernel density estimation (Turner & Sederberg, 2014). The estimated parameters for a given condition and participant are from the cell with the highest likelihood given the data. We apply this method separately to each participant and 9 experimental conditions (3 levels of time pressure for each of the 3 causal structures) to obtain the optimal parameters for each.

To make sure that the grid contains the optimal parameters we set a wide parameter range. The chain lengths were varied between [2, 4, …, 68, 70], which includes the chain lengths found previously for reasoning tasks (Davis & Rehder, 2020) and the range of number of samples people are generally thought to generate (Vul et al., 2014). For the Beta prior parameter, we first included values for *β* from 0 to 1 with step size 0.1. Next, we included values for *β* > 1 based on the principle that the range of priors should be symmetric about the uniform prior. That is, priors with *β* > 1 would need to differ from the uniform distribution as much as priors with *β* < 1. To achieve this, we computed the total variation distance (Levin & Peres, 2017) between the uniform distribution and each prior in the grid with a *β* < 1. Then we identified the set of *β* > 1 that had the same total variation distance. This procedure resulted in the following betas: *β* ∈ [0.1, 0.2, 0.3, 0.4, 0.5, 0.6, 0.7, 0.8, 0.9, 1, 1.11, 1.26, 1.45, 1.73, 2.14, 2.83, 4.14, 7.35, and 21.54]. For *β* = 0 the principle of symmetry about the uniform prior would lead us to pick *β* = ∞. However, as using a Beta(∞, ∞) prior would lead to responses only at 50%, we picked *β* = 100 instead.

In sum, we used a grid of 35 (values for the chain length parameter ranging from 2 to 70) by 21 (values for the *β* parameter ranging from 0 to 100) covering a wide range of plausible parameter values and simulated responses using (35 × 21 =) 735 different parameter combinations. While Davis and Rehder (2020) also estimated the causal parameters (base rates and causal strengths) of the causal structures that participants learned, we assume that participants learned the information they were presented accurately (we discuss this point further in the General Discussion). Hence with our setup the MS has only one free parameter (the chain length). The BMS has the *β* parameter for the symmetric Beta prior as a second free parameter.

#### Overall fit.

**Figure 5.**
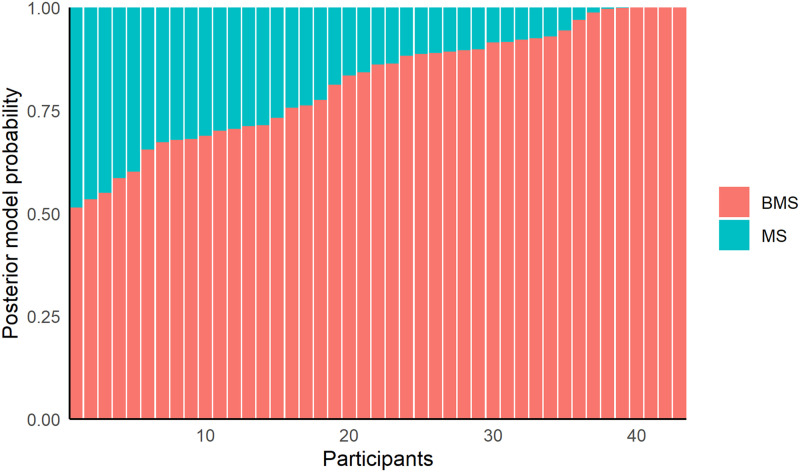
**Posterior model probabilities per participant for the BMS and MS.** Posterior model probabilities are approximated using BIC weights.

To quantify relative model performance of BMS to MS we computed BIC values for each set of fitted parameters (Schwarz, 1978). BIC, as compared to AIC, typically penalizes additional free parameters more strongly and so can be considered more conservative. We find that for 82.9% of the optimized models the BMS has a lower BIC value than the MS (mean Δ_BIC_ = −29.6). Next, we computed the average BIC weights per participant as approximations of posterior model probabilities (Neath & Cavanaugh, 2012; Schwarz, 1978; Wagenmakers & Farrell, 2004). We find that for each participant the BMS has a higher posterior probability than the MS (Figure 5).

#### Mean predicted judgments.

**Figure 6.**
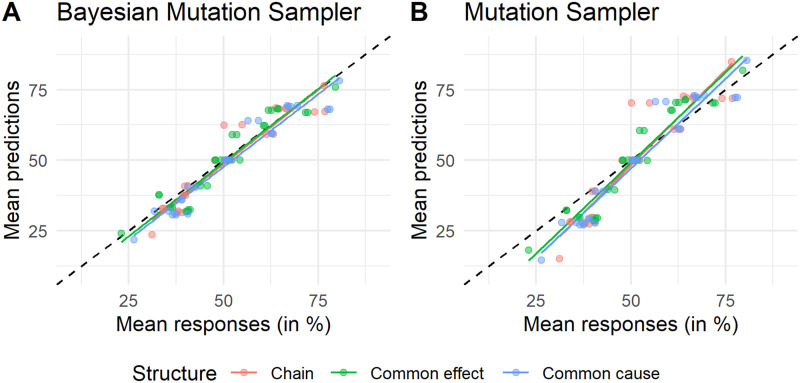
**Mean predictions of (A) the BMS and (B) the MS plotted against mean responses.** Dots and lines are colored based on the causal structure in the experiment. Each dot represents one of the 27 inferences. Each line represents a linear fit to the predicted and empirical means. Black dashed diagonal lines indicate error free predictions.

As discussed in the Introduction, the MS accurately predicts mean responses on a variety of causal judgment tasks. To assess the mean predictions of the BMS we computed the expected value of all predictions from the BMS with the best fitting parameters of each participant. Specifically, per inference and per causal structure, we computed the average across participants of the predictions at each percentage point, resulting in an averaged predicted distribution, and then computed the expected value of this distribution. We find that the predictions closely follow the observed mean responses (Figure 6), indicating that the BMS is a good account of mean responses. The BMS outperforms the MS in this regard (RMSE_BMS_ = 2.74; RMSE_MS_ = 7.51).

#### Variability of judgments.

In addition to mean judgments, another important behavioral index is the variability of judgments (Kolvoort et al., 2021). However, getting a reasonable estimate of the variability in judgments is often challenging as it requires the repeated elicitation of comparable judgments (see Kolvoort et al., 2021). To obtain such repeated measurements and to present results concisely a common practice is to collapse over symmetry in the causal networks (e.g., Davis & Rehder, 2020; Kolvoort et al., 2021; Rehder, 2018; Rottman & Hastie, 2016). The joint distribution of the causal networks in the experiment used here were highly symmetric, allowing us to collapse over the terminal variables (e.g., *P*(*Y* = 1|*X*_1_ = 1, *X*_2_ = 0) = *P*(*Y* = 1|*X*_1_ = 0, *X*_2_ = 1)), over the presence or absence of variables by flipping responses to the upper half of the response scale (e.g., *P*(*Y* = 1|*X*_1_ = 1, *X*_2_ = 1) = 1 − *P*(*Y* = 1|*X*_1_ = 0, *X*_2_ = 0)), and over unknown variables (e.g., *P*(*X*_1_ = 1|*Y* = 1) = *P*(*Y* = 1|*X*_2_ = 1)). In addition, since we did not find significant differences in parameters, we will collapse over response deadline conditions. Finally, we will collapse over the chain and common cause network structures as these have an equivalent underlying normative distribution. We do not use the common effect structure for this analysis nor for the analysis of distributions below, since the small number of observations would lead to unreliable estimates of variability. Collapsing resulted in 7 groups of inferences presented in Table 2 (see Appendix B for an overview of all inferences in each group).

**Table 2.** Grouping of inferences for variability and distributional analysis based on symmetry in chain and common cause causal network structures.

**Group**	**Inference type**	**Conditioning information**	**Queried variable**	**Normative response**	**Obs. per participant**	**Example**
1	Conflict	Two known variables with different values	1: terminal variable	75%	24	*P*(*X*_1_ = 1|*Y* = 1, *X*_2_ = 0)
2			2: middle variable	50%	12	*P*(*Y* = 1|*X*_1_ = 1, *X*_2_ = 0)
3	Ambiguous	Only one known variable	1: adjacent to known variable	75%	48	*P*(*X*_1_ = 1|*Y* = 1)
4			2: non-adjacent to known variable	62.5%	24	*P*(*X*_1_ = 1|*X*_2_ = 1)
5	Consistent	Two known variables with the same values	1: terminal variable	75%	24	*P*(*X*_1_ = 1|*Y* = 1, *X*_2_ = 1)
6			2: middle variable	90%	12	*P*(*Y* = 1|*X*_1_ = 1 *X*_2_ = 1)
7	Base rates	No known variables	–	50%	18	*P*(*X*_2_ = 1)

‘Queried variable’ refers to the variable that participants are asked to judge the probability of. ‘Terminal variable’ refers to either *X*_1_ or *X*_2_, and ‘middle variable’ to *Y* in Figure 1. The variable names in the example column refer to the variables as presented in Figure 1. See Appendix B for a full list of inferences in each group.

To index variability we use Gini’s Mean Difference (GMD; David, 1968; Yitzhaki, 2003), defined as the average difference between any two observations. We use this non-parametric index as judgments on causal reasoning tasks tend to not be normally distributed (Davis & Rehder, 2020; Kolvoort et al., 2021, 2022; Rehder, 2018; Rottman & Hastie, 2016). To compute the GMD of model predictions, we first computed averaged predicted distributions for each participant and inference group (by averaging the predicted distributions over the deadline conditions, the chain and common cause networks, and the different inferences in each inference group). We then drew 10,000 samples from these aggregated distributions which we used to compute the GMD.

**Figure 7.**
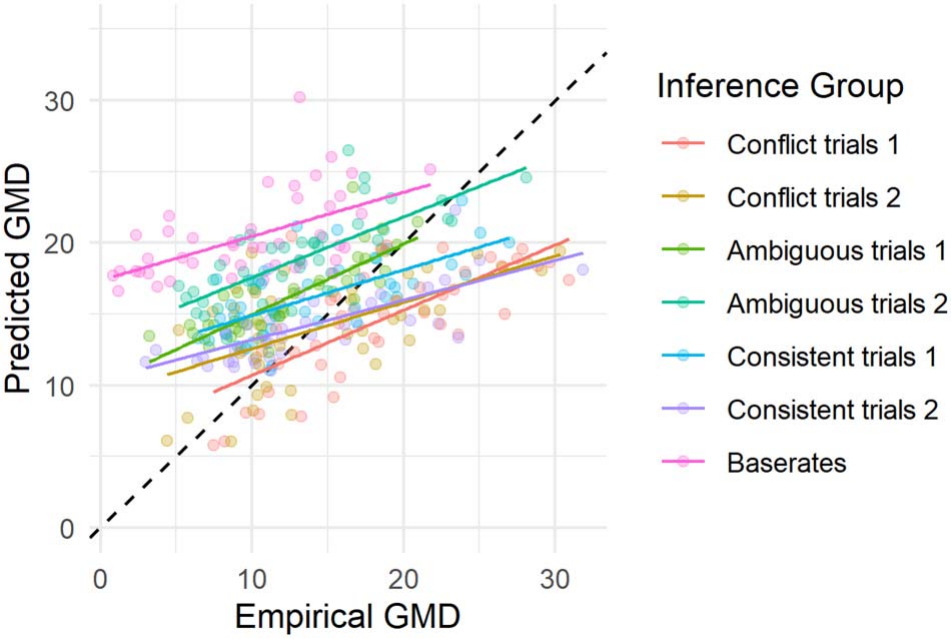
**Scatterplot of empirical variability and variability predicted by the BMS (indexed by Gini’s Mean Difference, GMD).** Responses and predictions are collapsed over the common cause and chain structures, the response deadlines, and into inference groups (see main text). Each dot represents one participant. Black diagonal indicates perfect predictions. Colored lines indicate mean linear trends per inference group.

We find an empirical mean GMD of 13.8 indicating there is substantial variability in responses. Both models predict mean variability to be higher (GMD_BMS_ = 16.4, GMD_MS_ = 19.2). The higher GMD for the MS is expected, because it predicts more extreme responses, increasing variability. Although the average variability of the BMS is higher than the observed variability, there are clear associations between the observed and predicted variability for each inference group (Figure 7 and Table 3). Table 3 presents the correlation coefficients of the predicted and empirical variability, for both BMS and MS.

**Table 3.** Pearson correlations of predicted and empirical variability as indexed by GMD.

	**R**	
	**BMS**	**MS**
Conflict trials 1	.72	−.19
Conflict trials 2	.58	.094
Ambiguous trials 1	.83	.4
Ambiguous trials 2	.74	.31
Consistent trials 1	.64	.37
Consistent trials 2	.68	.38
Base rates	.57	.38

Within inference groups, the BMS predicts differences in variability between participants (Figure 7). However, the model does not perform well at predicting differences in variability between inference groups. For instance, it consistently predicts base rate judgments to be more variable than they are, and it predicts that judgments in the conflict inferences 1 group are less variable than they are. That the BMS does not perform well in predicting between inference group variability might be due to that all different inferences are modelled with a single set of model parameters. While there seems no a priori reason that people use different priors for different inferences, it might be that the chain length differs based on the inference (see Gershman & Goodman, 2014; Hertwig & Pleskac, 2010; Vul et al., 2014; Zhu et al., 2020). When faced with a problem that is complex at first glance (e.g., an inference with conflicting conditional information), people could decide to sample for a longer duration. We return to this idea in the following sections. To get a better grasp of why some of the variability estimates are off we regard full response distributions next.

#### Distributions.

**Figure 8.**
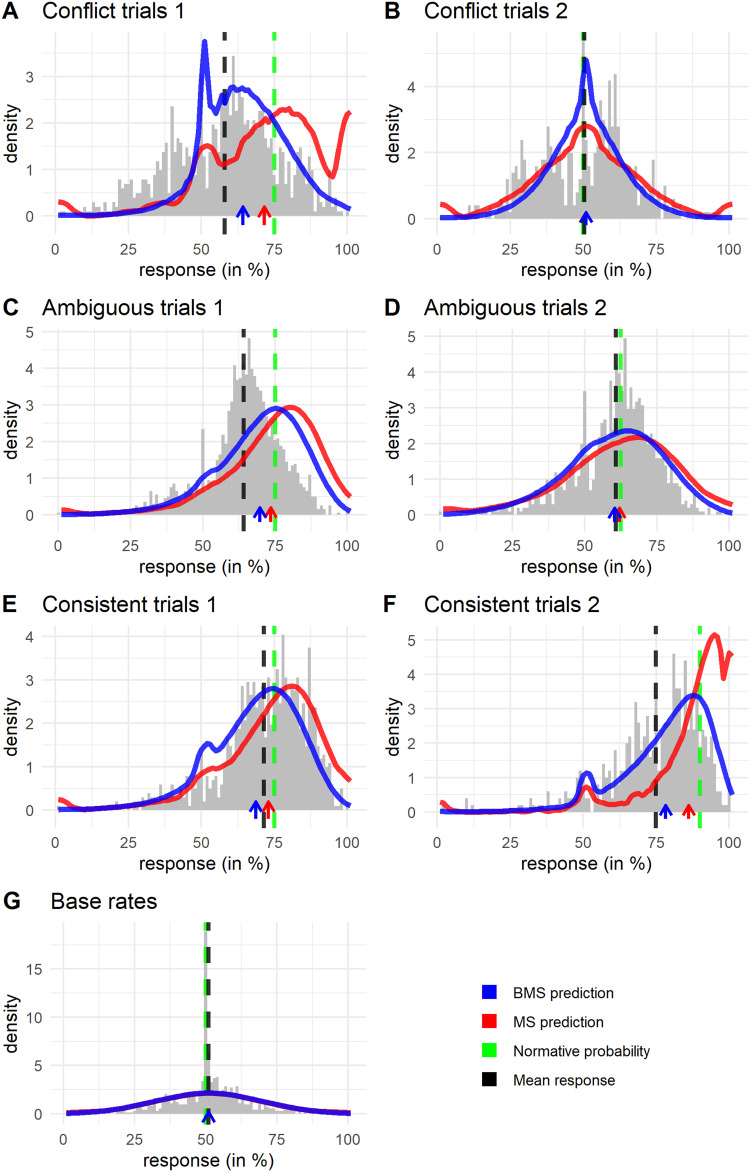
**Observed and predicted response distributions.** Blue and red solid-colored lines indicate predictions based on model fits from the BMS and MS respectively, the arrows on the *x*-axis indicate the mean prediction. Grey histogram represents participant responses with the black dashed line indicating the mean. The green dashed line indicates the normative probability.

To better understand the predicted distributions and how they match observed responses we present these distributions in Figure 8. Here the averaged best-fitting predictions of BMS and MS are presented together with histograms of participant responses.

First, let us discuss the distributional problems of the MS brought to light in the first part of this paper. The issue of extreme responses is visible relatively strongly in both the types of conflict trials (Figure 8A and B). This can be expected, since conflict trials (where the conditional information is conflicting), require the sampler to visit mixed variable states. These states are harder to reach for the sampler, since the sampler is biased towards consistent, prototypical states. Hence the probability of a default extreme response is higher. Some extreme predicted responses by the MS are also visible for the ambiguous and consistent trials (Figure 8C–F). The BMS does not produce any of these extreme responses. That there are fewer extreme responses for the MS than our analysis in the first part would indicate is because the chain lengths we found are higher than expected based on previous studies with the MS (Davis & Rehder, 2017, 2020). The second distributional issue we diagnosed of the MS, the lack of moderate conservatism, is also clearly visible in Figure 8 (panels A, C, E, and F). We see that the main mode of responses is at or more extreme than the normative probability. The BMS is more accurate in predicting where the bulk of responses are. Taken together, the BMS resolves the principal issues that the MS has in predicting where responses tend to be on the response scale.

The BMS is a clear improvement over the MS in predicting full response distributions and, according to our knowledge, the only process-level model of causal probabilistic reasoning that can satisfactorily account for response distributions. However, the predictions of the BMS are not perfect and certain limitations come to light when we regard the distributions for each inference type in more detail.

Figure 8A indicates that the BMS correctly predicts the mode of computed responses for conflict inferences where the middle variable is queried (Conflict trials 1). However, it wrongly predicts very few responses to be below 50%. Inspecting individual responses, we find that individual participants tended to respond on both sides of 50%. We observe this as well for the conflict trials where a terminal variable is queried (Conflict Trials 2, Figure 8B), hence it seems that the stimulus makes participants respond inconsistently due to the presence of conflicting cues. A substantial number of responses on this inference are possibly the result of random guessing which is not captured by the BMS. Such random responding might also explain why the frequency of responding at 50% is less than predicted. For the conflict trials where a terminal variable is queried (Conflict trials 2, Figure 8B) we see that the spike of responses at 50% is captured by the BMS prediction. What is not captured by the prediction is that participants tended not to respond close to 50%. One possible explanation for this observation is that participants rounded their responses to 50%. The reduced amount of responses near 50% occurs for both types of conflict trials, so it might be that the tendency to round to 50% is related to a participant’s uncertainty in their estimates (cf. Kolvoort et al., 2022).

For the ambiguous trials, we observe that the prediction seems to be quite accurate for inference group 2 (Ambiguous trials 2, Figure 8D), but for group 1 (Ambiguous trials 1, Figure 8C) participants are more conservative than predicted. Such a pattern could be explained if participants sampled less, i.e., used a shorter chain of samples, to form a judgment in response to stimuli in group 1 than for group 2. When chain lengths are shorter, the influence of the prior is stronger and so responses would be closer to 50%. In our modeling we fixed the chain length over inference types. However, previous research has indicated that it is possible that people adaptively change their desired number of samples as the estimated costs and benefits of further sampling are dependent on the problem type (Gershman & Goodman, 2014; Hertwig & Pleskac, 2010; Vul et al., 2014; Zhu et al., 2020). Why might participants use different chain lengths for these inferences? Remember that for these ambiguous trials the state of one variable is given, while the state of the other non-queried variable is unknown. For the first group of ambiguous inferences the given variable is adjacent to the queried one, and so these stimuli may be considered as less ambiguous than group 2, where the given and queried variable are separated by an unknown variable. The observation that responses to stimuli in first group (Figure 8C) seem less variable than the second group (Figure 8D) is congruent with this idea. Since the stimulus in group 1 is less ambiguous, participants might view it as easier and so obtain fewer samples to form a judgment. A repeated measures ANOVA indicates that response times are indeed significantly shorter for group 1 than for group 2 (*M*_1_ = 4.32 s, *M*_2_ = 4.62 s, *F*(39, 2891) = 14.73, *p* < .001, *BF* = 32.5), corroborating this explanation. This effect is not captured in our predictions as we did not model a process by which participants might decide to use different chain lengths.

For the consistent trials (Figure 8E and F) the BMS seems to capture the spread of responses rather accurately. For the base rate trials, however, the BMS severely underpredicts the spike of responses at 50% while capturing the rest of the variability quite accurately (Figure 8G). Compared to the other inference types participants have a strong tendency to respond at 50%. These base rate trials can be considered to involve the most uncertainty for participants compared to other inferences, since no conditioning information is provided. This uncertainty could lead to default responses or ‘guessing’ at 50% (Kolvoort et al., 2021, 2022; Rottman & Hastie, 2016). Upon viewing the stimulus participants might forego on sampling and instead respond at 50%. This would indicate a mixture of processes, where prior to the sampling process one might decide not to sample and instead respond in a default fashion. As we did not specify any mixture of processes the large spike at 50% is not captured by the BMS predictions here.

It is important to realize that we are putting up a very high bar when considering the ways in which the predicted distributions do not exactly match participant responses. The modeling of full response distributions is a complex endeavor as there are many processes and mechanisms that likely affect distributions of judgements which traditionally would be filtered out by taking the mean. In fact, many of the limitations of the BMS predictions just discussed point towards the need to specify additional processes to match the empirical distributions more accurately. We return to this point in the General Discussion, before doing so we first regard the estimated parameter values.

#### Estimated parameters.

A summary of fitted BMS parameter per response deadline condition is shown in Table 4. The overall median *β* parameter was 1.45, which is close to the uniform distribution (*β* = 1) that is often considered the prototypical uninformative prior. For most participants (79.1%) the mean *β* is larger than one, indicating they expected answers to be closer to 50%, validating our choice to include values for *β* > 1 (cf. Zhu et al., 2020). Higher values for *β* lead participants to be more conservative in their responses. This could explain (a part of) the substantial conservatism observed on this (Kolvoort et al., 2022) and other causal reasoning tasks. While no participant was fitted best by the upper bound of the grid (*β* = 100), we find that in 5.94% of cases the best fitting *β* is zero, the lower bound of the grid. This indicates that in only a small subset of cases the relative frequency method of generating judgments (as proposed by the original MS) was used. As could be expected the prior used was not affected by response deadlines (*F*(2, 336) = 1.04, *p* = .433, *BF*_H1_ = 0.036).

**Table 4.** Summary of fitted BMS parameters based on fitting to each participant, structure, and deadline condition separately, resulting in 387 sets of parameters.

**Deadline**	***β* parameter**			**Chain length**		
	**Median (*SD*)**	**Minimum**	**Maximum**	**Median (*SD*)**	**Minimum**	**Maximum**
6 s	1.73 (2.58)	0	21.5	40 (19.0)	8	70
9 s	1.11 (2.77)	0	21.5	46 (19.5)	4	70
20 s	1.45 (3.14)	0	21.5	50 (20.4)	6	70
Overall	1.45 (2.83)	0	21.5	44.9 (19.7)	4	70

The *β* parameter refers to the fitted Beta(*β*, *β*) priors.

The average chain lengths we find fall within a range expected based on the literature. Zhu et al. (2020) for instance found best fitting chain length for certain participants to be well over 200 in simple probability judgment tasks. While Davis and Rehder (2020) found a maximum mean chain length of 28 for causal inference studies, the average best fitting chain length for some causal intervention or categorization studies was above 60. There seems to be a trend of increasing chain lengths for longer deadlines (Table 4). This would be consistent with the hypothesis that participants sample longer when they have more time to respond. However, a repeated measures ANOVA indicates that there is no statistical support for such an effect (*F*(2, 336) = 3.74, *p* = .121, *BF*_H1_ = 0.25). 14.0% of fitted chain lengths reached the maximum value of 70. This reflects that larger chain lengths are more difficult to estimate, since the differences in the predictions of the BMS become increasingly smaller as chain lengths increase (Appendix A). While there is some uncertainty regarding the exact values of the higher chain lengths, the median chain lengths we find are noticeably higher than found by Davis and Rehder (2020) for experiments involving causal inference, as they found the best fitting chain lengths to range from 4 to 28 in these types of tasks^7^.

#### BMS and behavioral measures.

Lastly, we studied the relationship between the fitted parameters and other behavioral measures to validate the BMS. As the BMS is a process model, it should relate to behavioral measures not specified in the model itself. We looked at three important behavioral measures: response times, accuracy, and conservatism. Response times here are especially of interest as they are not part of the data used to fit the BMS. Accuracy was defined as the absolute distance of responses from the normative answer and conservatism was defined as the absolute distance of responses from 50%. For ease of interpreting the statistics, we multiplied both accuracy and conservatism values with minus one, as by doing so higher values indicate higher levels of accuracy and conservatism respectively. We computed the mean fitted parameters and the means of these behavioral measures over all conditions per participant and tested their relationships using Spearman correlations (Table 5).

**Table 5.** Spearman correlations of mean fitted parameters and behavioral measures per participant.

	**Response time**	**Accuracy**	**Conservatism**
Chain length	.344*	.893***	−.323*
	(3.29)	(>100)	(2.48)
*β*	−.0974	.00740	.749***
	(0.405)	(0.341)	(>100)

See main text for definitions of Accuracy and Conservatism. Numbers in brackets refer to Bayes factors (BF_H1_, i.e., for the existence of a correlation), computed using the Bayes Factor package in R using default settings (Morey & Rouder, 2014). Asterisks indicate *p*-values: *p < .05, **p < .01, ***p < .001.

We find that chain length is positively correlated with response times and strongly with accuracy, while being negatively correlated with conservatism. These correlations all reflect a higher task performance of individuals that sample for a longer duration: Firstly, as the chain length is a direct reflection of the sampling duration, a correlation with response time is expected. Secondly, longer chain lengths indicate more computed responses that get to the normative answer as the effect of the starting point bias decreases, resulting in higher accuracy. Finally, longer chain lengths also imply less influence of the prior, which is reflected in responses being less conservative. This is also reflected by the strong positive correlation between the *β* parameter and conservatism. Higher values of *β* imply the use of a prior that has more probability mass near 50% which results in more conservative responses. In sum, these correlations support the BMS model specification by showing expected relations between the parameter estimates and behavior.

## GENERAL DISCUSSION

The aim of this paper was to understand distributions of probabilistic causal judgments. In the first part we diagnosed problems with the distributions of responses that a process model of causal reasoning, the Mutation Sampler (MS), predicts. The two main problems we identified were that the MS predicts a non-trivial number of extreme responses at 0 or 100 %, and that it predicts the bulk of computed responses to be centered near the normative probability. Contrary to these predictions, data indicate that people actually refrain from using the extreme ends of the response scale and that the bulk of their responses tends to lie in between the normative answer and 50% (Kolvoort et al., 2021, 2022; Rehder, 2018; Rottman & Hastie, 2016). We traced these issues back to the process by which the MS forms judgments based on samples and proposed to extend the MS by the incorporation of prior information into judgments. In the second part of the paper, we formalized the idea of incorporating prior information into judgments and presented the Bayesian Mutation Sampler (BMS). The BMS combines the sampling process of the MS with the use of prior information. We fitted the BMS and MS to data from a recent causal reasoning experiment to illustrate that the BMS provides a better account of the data, and found that the BMS resolves the distributional problems associated with the MS. Although the BMS predictions are not perfect, the model is able to account for a lot of the variability we observe in causal judgments. To our knowledge the BMS is the only computational (process-level) model of causal reasoning that is able to capture response distributions to this degree. This is not an easy feat, especially considering that the model uses only two free parameters to predict full response distributions for multiple inference types. These findings provide evidence for the notion that the variability observed in causal judgments is due to a sampling mechanism. This is in line with findings implicating sampling being the source of variability in children’s responses on causal learning tasks (Bonawitz et al., 2014)

Our formulation of the BMS entails some important theoretical commitments. We proposed that participants have prior beliefs regarding likely (conditional) probabilities and that they incorporate these beliefs into their judgments in Bayesian fashion. Amongst other reasons, we motivated the use of priors by the observation that participants do not provide extreme judgments. That is, we interpret the avoidance of the extremes of the response scale as a rational adjustment to small sample sizes via the incorporation of prior information. However, there are other plausible explanations for the observation that people avoid making extreme judgments. One option would be that people avoid the extremes of the response scale due to a response bias; e.g., participants could be reluctant to express the confidence that a judgment of 0% or 100% might imply (e.g., DuCharme, 1970; Phillips et al., 1966). Another option could be that people use a particular mapping of objective probabilities to subjective probabilities, such as a probability-weighting function used in Prospect Theory (Tversky & Kahneman, 1992). The BMS posits that people avoid the extremes of a probabilistic response scale due to their beliefs (encoded by priors) instead of this being the result of a particular mapping from beliefs to a response scale. While we are not necessarily committed to the idea that participant beliefs map straightforwardly onto a probabilistic response scale, we did not implement a mapping function.

A different approach was used in the original MS paper, where the authors used a scaling factor to map the probabilities computed by the MS to the response scale (Davis & Rehder, 2020). A free ‘scaling’ parameter *s* was used such that a predicted response = *s* * *p*, where *p* is the probability generated by the MS. The MS combined with such a mapping function can produce responses that fall outside the response scale and so we did not consider it to be a proper account of the variability in causal judgments (Appendix C provides an illustration of this effect). Including such a scaling parameter improves the fit of the MS, but it is still outperformed by the BMS (Appendix C). Moreover, as the use of a (Beta) prior is theoretically motivated the BMS is clearly the favored model.

We can consider the alternative explanations for conservatism mentioned earlier as other theoretically motivated mechanisms that could map probabilities produced by the MS to a response scale. In other words, a response bias related to a reluctance to express confidence or the use of a probability-weighting function as in Prospect Theory could be assumed instead of a Beta prior. These explanations could predict that responses are ‘pushed’ towards the middle of the response scale in a similar way as the incorporation of a symmetric prior does. Due to this we cannot use the distribution of responses in the current experiment to distinguish between these explanations. One possible approach to empirically verify the prior mechanism proposed by the BMS would be to conduct an experiment in which one manipulates participant beliefs about what the likely answers to a causal probabilistic query are. To manipulate prior beliefs participants need to be presented with data. This data could be of various forms, such as training data that imply extreme probabilities, fabricated responses from other participants, or participants could be provided feedback on their own responses. If the BMS is correct we would expect participants to update their prior in light of this new data which in turn would systematically affect their judgments. We suggest future research to run such experiments to confirm the use of priors. As it currently stands, though, we maintain that the use of priors is the most plausible explanation of the observed behavior as there are compelling arguments in its favor that go beyond response mapping. There are the usual normative arguments in favor of a Bayesian approach and people have been shown to reason in a Bayesian manner (that is, using priors) in many other domains (see Oaksford & Chater, 2020; Parpart et al., 2018; Vul et al., 2014; Zhu et al., 2020).

In all, our work contributes to a recent movement in the field of causal reasoning arguing that that the variability observed on tasks reflects information of interest (Kolvoort et al., 2021; O’Neill et al., 2022). Understanding the variability in responses and modeling full response distributions can be a challenging task but it comes with important benefits (see O’Neill et al., 2022). To promote future research in this direction the remainder of this paper discusses potential pitfalls and gains of such an approach and suggest promising directions of research into causal judgments.

### The Importance of Distributions

Important benefits of shifting the explanatory focus from mean responses to response distributions include the potential to ask more questions and providing safeguards against drawing erroneous conclusions.

#### Asking more questions.

Recent examples of using response distributions to better our understanding of causal reasoning include initial preliminary investigations by Rehder (2018) and Rottman and Hastie (2016), and the more recent studies by Kolvoort et al. (2021) and O’Neill et al. (2022). Using a novel experimental design Kolvoort et al. (2021) elicited repeated causal judgments which allowed them to establish the presence of substantial within-participant variability that differs per inference type. O’Neill et al. (2022) analyzed response distributions of existing and new vignette-based experiments which led them to conclude that causal judgments are often graded and multimodal. This result allowed them to assess theories of causal reasoning and suggest improvements based on a graded concept of causation. Similarly in our work, both predicted and empirical distributional phenomena informed the development of the BMS. The observation that the MS predicts extreme responses and many responses near the normative probability was crucial in determining how the MS could be improved. A shift towards analyzing distributions will allow researchers to target more behavioral phenomena and subsequently develop more comprehensive theories.

#### Stop erroneous inferences.

**Figure 9.**
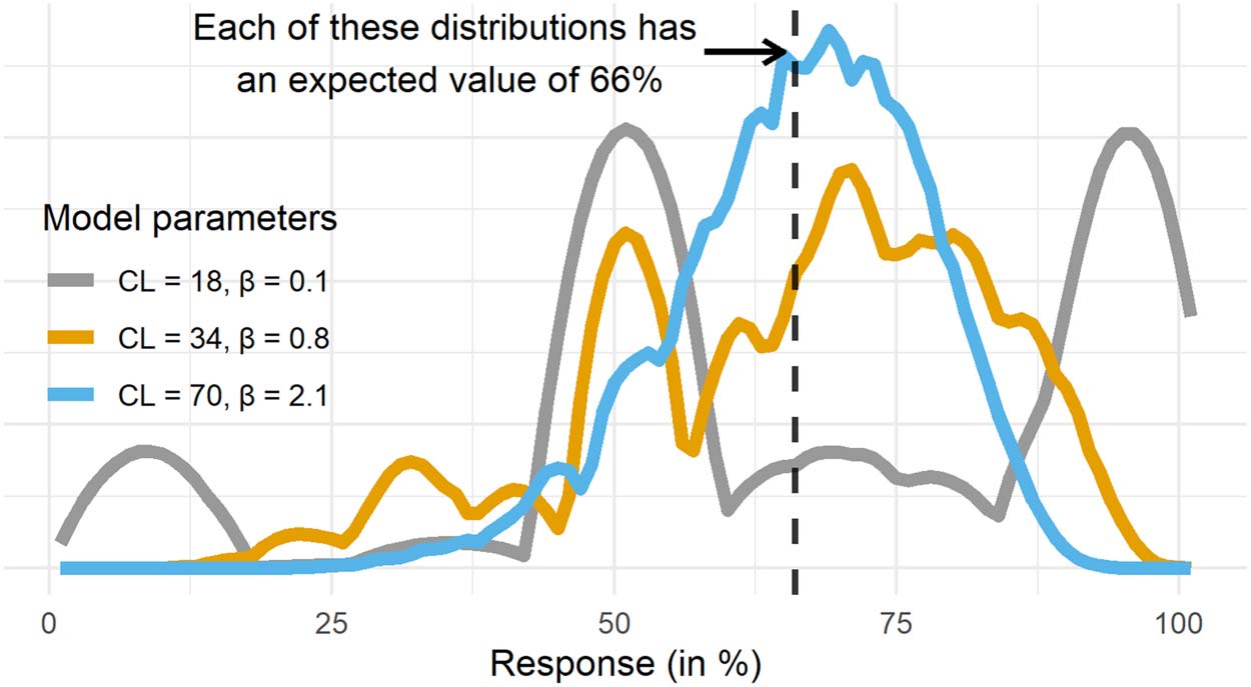
**Illustration of BMS predicted response distributions of the inference *P*(*X*_1_ = 1|*Y* = 1, *X*_2_ = 0) for 3 different sets of parameters using a chain causal structure with the same parametrization as used in the experiments studied in this manuscript.** 10,000 simulations were run to compute each of the three distributions. Each of the distributions was smoothed using a kernel density estimate as specified by the PDA method. CL refers to the chain length parameter, *β* refers to the parameter for the Beta(*β*, *β*) prior. The vertical dashed line indicates the expected value of all three distributions.

In addition to leading us to more questions, using distributions instead of means as our explanatory target can help us to not draw erroneous conclusions. It has become clear from the current work and recent investigations that the variability of causal judgments does not just reflect noise and that these response distributions are often multimodal and non-normal (Kolvoort et al., 2021; O’Neill et al., 2022; Rottman & Hastie, 2016). This entails that what we infer from statistical and cognitive models which characterize only mean responses can be severely misleading. This can be illustrated by looking at what would happen if we had merely modeled mean responses using the BMS. Figure 9 shows three predicted distributions for a single inference of the BMS using different sets of parameters. While the chain lengths range from 18 to 70 and the *β* parameter from 0.1 to 2.1, the mean response for these distributions is the same. Hence the model would not be identified if we were to only regard mean responses. Moreover, these distributions, though having the same mean, imply a very different type of responding (cf. Anders et al., 2016). For instance, while it is common to assume that most responses are near the expected value of a response distribution, Figure 9 shows that the BMS can produce varying densities of responses near the expected value while keeping the expected value itself fixed. This is an issue related to model identifiability (cf. van Maanen & Miletić, 2021) and it is quite common outside of the domain of causal reasoning (for instance with models predicting response time distributions, e.g., Anders et al., 2016). For the BMS, increasing the chain length moves the expected value towards the normative response, while increasing *β* moves the expected value of the predicted distribution towards 50%. Hence for any percentage point *Z* that is between the normative probability and 50%, there are an infinite number model parameter combinations that would produce distributions with the expected value at *Z*. Due to this solely focusing on the mean response can lead to drawing multiple erroneous conclusions. One wrong conclusion could be that most responses lie near the mean predicted response, which is often not the case for causal judgments (e.g., O’Neill et al., 2022). Relatedly, the amount of disagreement between participants could be overestimated if one were to erroneously conclude that the grey prediction in Figure 9 accurately captures group-level responses (compared to other predictions in Figure 9).

To make sure researchers do not draw erroneous conclusions based on models that overemphasize the importance of the central tendency of responses, O’Neill et al. (2022) recommend to plot histograms of response data regularly and to assess whether the underlying distributional assumptions of their (statistical) models are met. This latter point is important, as the standard linear models often assume equal variances over participants and conditions, while we now know that this assumption is often violated in causal judgments (e.g., Kolvoort et al., 2021; O’Neill et al., 2022).

#### Using generative models to target distributions.

Another recommendation O’Neill et al. (2022) give is for researchers to move towards modeling response distributions using a generative approach. This computational approach is becoming more widespread in psychological and brain sciences (see for recent overviews, Ahn & Busemeyer, 2016; Forstmann et al., 2016; Guest & Martin, 2021; Jarecki et al., 2020; Lee et al., 2019; Ratcliff et al., 2016; Turner et al., 2017; Wilson & Collins, 2019). Generative modeling involves constructing computational models that embody theoretical assumptions about how behavior is generated (Haines et al., 2020). This involves characterizing the psychological process that turns inputs (stimuli) into outputs (behavior or judgments) in mathematical terms. The BMS (as well as MS) is a generative model of the process by which people generate causal probabilistic judgments by way of sampling from a causal network (Davis & Rehder, 2020).

Generative modeling naturally leads researchers to focus on psychologically interpretable parameters (such as the number of samples or type of prior information) instead of on estimating descriptive effects. Such descriptive effects are often defined using differences in mean response between conditions and tested using standard parametric statistical tools (such as *t*-tests, regressions, ANOVAs, etc.). However, we know that means often don’t capture response distributions in a meaningful way and that this violates the assumptions of the standard parametric tests (see Haines et al., 2020). These descriptive effects could also be modeled using ‘descriptive’ models that predict only mean responses (e.g., Rehder, 2018; Rottman & Hastie, 2016), but this could lead one to draw erroneous conclusions as the mean response could misguide researchers (see Figure 9).

Instead, generative modeling helps researchers to account for more than just averaged behavior. When mathematically specifying the data-generating process a researcher has to incorporate assumptions about the psychological processes that generate empirical data. These assumptions should allow the model to generate predictions that mimic not just empirical means, but empirical distributions.

While generative models can be used to characterize group-level behavior, often they are implemented at the level of individual psychological processes. This allows for the fruitful study of individual differences, which could help in assessing competing models of causal reasoning (a good example can be found in Gerstenberg et al., 2021). We did not focus on explaining such individual differences here, but the BMS can in principle explain inter-individual variation by appealing to differences in how long people sample and the prior information they use. Since it is a longstanding question in the field of causal judgments to what extent the observed variability is due to within- or between-participant variability (Davis & Rehder, 2020; Kolvoort et al., 2021; Rehder, 2018; Rottman & Hastie, 2016), this seems to be an important direction for future research.

Related to individual differences, note that Davis and Rehder (2020) did not fix the causal parametrization of the causal network in the MS, instead estimating the causal parameters (i.e., base rates of the causal variables and causal strengths) to account for participants not learning an accurate representation of the causal network. Consequently, there is no strict one-to-one correspondence between their results and ours. The justification for estimating causal parameters was that we cannot assume that participants’ representations actually conform to what they are taught during the experiment about the causal networks. There is some force behind this argument as it is unlikely that participants learn the qualitative and quantitative aspects of the causal systems exactly as how they are presented to them. However, the current study was not aimed at understanding such individual differences. As mentioned, the current implementation of the BMS could explain individual differences by appealing to differences in how much people sample and the prior information they use. It might be that other individual factors need to be estimated to capture individual differences, such as individually learned causal parameters or, for instance, individual subjective probability weighting functions (Tversky & Kahneman, 1992) or differences in the order of processing of variables (Mistry et al., 2018). Which factors explain individual differences in probabilistic causal judgments remains an open question for now.

### Overcoming Challenges in Studying Distributions: Extending the BMS

The study of full response distributions, possibly using generative process models, is a promising direction and will help advance our understanding of the cognitive processes responsible for causal judgments. This type of work does, however, come with its own set of challenges.

#### Modeling the multitude of processes resulting in a response.

One possible direction to improve the BMS would be to adapt its core features. It could be that a different sampling mechanism (e.g., a Gibbs sampler instead of the MH algorithm; cf. Davis & Rehder, 2020) or differently shaped prior could help explain more of the variability. However, our results seem to indicate that the limitations of the BMS predictions are due to additional processes at play. That is, to capture empirical response distributions more accurately we need to model these processes.

The existence of additional processes affecting responses is a big challenge in trying to account for response distributions. There are a multitude processes that affect how judgments are made. Examples of such processes are the rounding of estimates (e.g., Budescu et al., 1988; Costello & Watts, 2014; Erev et al., 1994; Kleinjans & van Soest, 2014), guessing (Kolvoort et al., 2022; Kong et al., 2007; Schnipke & Scrams, 1997), a general mixture of multiple problem-solving strategies (Archambeau et al., 2022; Evans, 2008; van Maanen et al., 2014, 2016) and ‘dynamic effects’ such as fatigue, boredom, and learning (e.g., Gunawan et al., 2022). All these processes affect participant’s judgments on experimental tasks and partly determine the resulting response distribution.

The existence of all these additional processes is an important rationale in studying mean judgments: averaging across many trials filters out supposed noise. However, when targeting response distributions with generative models this solution is not available. Instead, we need to tackle the inherent complexity and develop theories and models explaining the target distributional phenomena.

While there are many processes that can be modelled, we focus here on two that our results indicated might play a large role in determining the observed distributions. The first is using a mixture of strategies. One likely strategy that is consistent with our observations is a guessing strategy. We observed varying peaks of responses at 50% which have been attributed to guessing before (Kolvoort et al., 2021, 2022; Rottman & Hastie, 2016). A second alternative strategy is that of adapting chain lengths based on the inference type one is confronted with. Such a mechanism has been suggested before (e.g., Zhu et al., 2020) and could, for instance, explain the difference in fit between the two types of ambiguous inferences (see Figure 8). While there can be many other processes affecting responses, we hypothesize that these two are likely to account for a substantial amount of the unexplained variability.

#### Modeling mixtures.

People can employ a variety of strategies to perform a particular task and so it is often assumed that observed behavior is the result of a mixture of such strategies (Campbell & Xue, 2001; Coltheart et al., 2001; Couto et al., 2020; Donkin et al., 2013; Dunlosky & Hertzog, 2001; Dutilh et al., 2011; Evans, 2008; Kahneman, 2011; Smith et al., 1998; van Maanen et al., 2014; van Maanen & van Rijn, 2010; van Rijn et al., 2003). For example, the dual-process framework of decision-making is built on the idea we can solve problems in either a more intuitive/heuristic manner or in a more deliberative manner (Evans, 2008; Kahneman, 2011; Stanovich, 1999). Intuitive reasoning is characterized by being automatic, fast and non-conscious, which is contrasted with deliberative reasoning that is more rule-based, slow, analytic and controlled. We can see this in two ways related to the BMS. One way is to consider the BMS to implement both intuitive and deliberative reasoning. If sampling chains are short responses are most affected by the biased starting points (prototypes), which can be seen as intuitive. When sample chains are long, the response is based more on the learned statistical relationships and we can consider this deliberative. Another way to look at this is to consider all sampling (e.g., as implemented by the BMS) as a deliberative way of reasoning. In this case, intuitive reasoning would be implemented by a wholly different mechanism. For instance, a more heuristic manner by which participants could respond on causal reasoning tasks would be by using a simple ‘tallying’ rule (Gigerenzer & Gaissmaier, 2011; Kolvoort et al., 2022; Rottman & Hastie, 2016), which involves counting positive cues (present variables) and subtracting negative cues (absent variables) to form a judgment. Even simpler strategies would be to just guess 50% or respond randomly, which is believed to happen on a subset of trials (Kolvoort et al., 2022; Kong et al., 2007; Schnipke & Scrams, 1997).

There is evidence that participants use such simple strategies in causal judgment tasks. One salient feature of response distributions in the probabilistic causal judgment literature are spikes of responses at 50% and it has been found that the size of these spikes differs per inference type (Kolvoort et al., 2021; Rottman & Hastie, 2016). Our results are in line with these findings. We found spikes of responses at 50% whose size depended on the inference type (see Figure 8). The large spike at 50% for base rate judgments stands out the most as it is substantially larger than the BMS would predict (Figure 8G).

We believe that this large spike at 50% for base rate judgments may reflect guessing or a ‘default’ response at 50%. In fact, spikes at 50% have previously been associated with uncertainty in the response (Kolvoort et al., 2022; Rottman & Hastie, 2016). When participants are uncertain or do not know how to respond they could employ their default response strategy and respond at (or around) 50%. This reasoning might explain why the spike at 50% is highest for base rate trials. For base rate trials the stimulus is most uncertain, as no information regarding the other variables in the causal structure is given. Hence based on this uncertainty explanation we would expect to see many such 50% responses for base rate queries.

While our modeling approach assumed that all responses were generated by way of mutation sampling, it is likely that participants varied in how they responded and that in a subset of trials they just guessed 50%. Additionally, for Conflict trials 1, in which the queried variable is adjacent to two opposing cues, we found that individual participants responded on both sides of 50%. This type of responding is consistent with a strategy of random responding.

If indeed the observed response distributions on causal judgment tasks are the result of a mixture of strategies, then it appears that the mixture proportions are dependent on the inference type. One piece of evidence for this is that we found substantially larger spikes for base rate inferences. More evidence come from previous studies, both Rottman and Hastie (2016) and Kolvoort et al. (2022) concluded that the frequency of 50% responses depended on the uncertainty that reasoners might have. That is, they found spikes to be smallest for consistent inferences, larger for ambiguous inferences, and largest for inconsistent inferences. We also observe this pattern in our data (Figure 8). These findings seem to point toward a mechanism in which people resolve their uncertainty by responding using the middle of the response scale (Kolvoort et al., 2022). It suggests that people adapt their response strategy based on an uncertainty-related feature of the stimulus. People use the ‘guessing strategy’ more often when there is a lot of uncertainty (e.g., a conflict or base rate trial) versus when there is not (e.g., a consistent trial).

#### Adaptive chain lengths.

Another potential source of variability that comes to light once we start modeling the full response distributions is that possibility of variable chain lengths. That is, it may be that the number of samples one considers for making a judgment, could differ per inference type. We found that for the ambiguous trials, where the state of one variable was unknown, participants were more conservative and responded more quickly when the known variable was adjacent to the queried one. This is consistent with the effects of having a shorter chain length. In the Results section we proposed that this might be due to these inferences being less ambiguous leading to people thinking they can be relatively accurate without needing to generate many samples.

While we could estimate the chain length separately for each inference type, a more principled approach would be to determine why and how chain lengths differ and to incorporate this into the BMS. Future research could focus on investigating this relation between stimulus and chain length. Zhu et al. (2020) suggest that how much someone samples could be dependent on problem complexity. This would be consistent with our ambiguity-based explanation. Related to this idea, other researchers have proposed an adaptive scheme in which the costs and benefits of samples are weighed to determine how many samples to generate (Gershman & Goodman, 2014; Hertwig & Pleskac, 2010; Vul et al., 2014; Zhu et al., 2020). These seem like fruitful ways to extend the BMS.

## CONCLUSION

We studied the predictions of the MS, as it currently is the most promising process-level model of causal reasoning, and found it has some shortcomings in explaining full response distributions. The original MS model was developed to implement four psychological principles that apply to causal inference: we think about concrete cases, we make small adjustment to these cases, we have a bias for prototypes, and we can only draw a limited number of samples. By developing the BMS and showing its improved performance we have argued for an additional principle to be added to this list: people make use of prior information. By adding this principle, and implementing it with the BMS, we showed it is possible to account for more distributional phenomena in causal judgments. We hope this work spurs other researchers to focus efforts on analyzing more than just mean responses as we believe this will improve our understanding of underlying cognitive mechanisms greatly.

## AUTHOR CONTRIBUTIONS

Ivar R. Kolvoort: Conceptualization; Formal analysis; Methodology; Writing—Original draft; Writing—Review & editing. Nina Temme: Formal analysis. Leendert van Maanen: Conceptualization; Methodology; Writing—Original draft; Writing—Review & editing.

## FUNDING INFORMATION

This work was supported by an Interdisciplinary Doctoral Agreement grant awarded to Leendert van Maanen by the University of Amsterdam.

## DATA AVAILABILITY STATEMENT

The experimental data analyzed during the current study are available in the OSF repository of the original experimental study at https://osf.io/bz9vj/. Model code and helper functions to run simulations with the BMS and MS are available at https://osf.io/xd9az/.

## ETHICAL APPROVAL

The study was approved by the local ethics committee of the University of Amsterdam (nr. 2019-PML-10019).

## Notes

^1^ Also known as causal graphical models, graphical probabilistic models, or causal Bayes’ nets.^2^ The chain and common cause structure had the same causal parameters, with base rates of .5 for all variables. The effects in the network had a probability of 75% when their parent was present and 25% when it was not. In the common effect structure, the two causes combined by way of a Noisy-OR gate (Cheng, 1997) with causal strengths of 50% and with base rates of 50%. This meant that the effect had a 0% probability if no causes were present, 50% when one cause was present, and 75% when both causes were present (hence the base rate was .43 for the effect).^3^ Davis and Rehder (2020) implemented this mechanism by initializing the number of visits to each network state with 10^−10^. When the required states for an inference are not visited Equation 1 then simplifies to 10^−10^/[10^−10^ + 10^−10^] = .5.^4^ The joint distribution of the causal variables also ties into this, since if there is a network state that has a small normative probability, it will be harder for the sampler to reach as well. This makes it that we observe the largest peaks in Figure 2A, where the query refers to a state where *Y* = 1 and *X*_2_ = 0, an unlikely state.^5^ One can interpret 50% responses as ‘extreme’ conservatism.^6^ While conceptually different, our approach is computationally equivalent to one which would assign a prior probability to all the possible network states instead of to likely correct responses to queries. That is, if we would add *β* visits to all system states in Equation 1 we would get Equation 2.^7^ The uncertainty regarding the exact values of higher chain lengths does not affect the conclusion that we found higher chain lengths, since if participants indeed used only few samples (smaller chain lengths) to make judgments, our parameter recovery study indicates we would have recovered those parameter values accurately (Appendix A).
