# Supplementary material for: The Bayesian Mutation Sampler Explains Distributions of Causal Judgments
Source: Open Mind (Camb). 2023 Jun 15;7:318–49. doi: 10.1162/opmi_a_00080 (PMC10320818; doi:10.1162/opmi_a_00080)
Supplement: Supplementary file 1 [file opmi-07-318-s001.pdf]

Appendix A: Parameter recovery study

To assess whether the Bayesian Mutation Sampler (BMS) is identifiable and what the best way of fitting it to data is we conducted a parameter recovery study. We simulated data using the BMS and then fitted the BMS to the simulated data to test whether the fitted parameters are similar to those used for simulating the data.

We test two methods of fitting the BMS to data: (1) a method employing a traditional iterative optimization approach, and (2) a method that uses a two-step grid search. Both these methods make use of the PDA method to compute 'synthetic' likelihoods (See main text; Holmes, 2015; Turner & Sederberg, 2014). Noteworthy is that each of these methods make use of the fact that the BMS has a strongly restricted parameter space, i.e. it has only two free parameters and one of those (the chain length) is an integer.

To assess the extent to which the fitting methods recover the simulated parameters we compute correlations between the true and fitted parameters. We will deem correlations below .5 to be poor, between .5 and .75 to be fair, between .75 and .9 to be good, and above .9 to be excellent, similar to criteria used in other parameter recovery studies (e.g. Anders et al., 2016; van Maanen et al., 2021; van Ravenzwaaij & Oberauer, 2009; White et al., 2015).

Simulating data

To simulate data we picked chain length parameters from a range of 2 to 50 and β parameters from a range of 0 to 15. We randomly sampled 50 values for each of the parameters from a uniform distribution over their ranges and randomly paired these. In this way we obtained 50 unique combinations of the chain length and Beta parameters which were used to simulate data.

Datasets were simulated using the causal parameters (i.e. base rates and causal strengths) of the experimental study that we fitted the BMS to in the main text (Kolvoort et al., n.d.; these are the same causal parameters as used in Experiment 1 by Rottman & Hastie, 2016). That is, we simulated data separately for the for the Common Cause/Chain network (these had equivalent parametrizations) and for the Common Effect network.

Next, we simulated datasets with 27 and 54 observations per parameter combination, reflecting either 1 or 2 observations per inference per participant (there are 27 different inferences in the experiment by Kolvoort et al.). The smaller dataset has the same number of observations per parameter combination as the empirical data that we fitted the models to in the main text has per participant. Hence if we find good recovery for these smaller datasets, we can be confident in the parameters we obtain from fitting the models to the empirical data.

In all we simulated four data sets, two for each of the causal network structures with either 27 or 54 observations per parameter combination.

Method 1: iterative optimization of Beta prior parameter for each chain length

The first method we test can be considered more traditional as it uses an iterative method to find the optimal β parameter. In a first step we optimized the β parameter for each possible chain length, that is for each integer from 2 to 50. This optimization was done using the PDA method (Holmes, 2015; Turner & Sederberg, 2014) and the base R function *optimize*, which uses an iterative method combining golden-section search and successive parabolic interpolation (Brent, 1973; R Core Team, 2019). In this way we end up with the best fitting β parameter for each possible chain length. In the next step we simply pick the chain length and optimized β parameter that maximize the summed likelihood.

Method 2: two-step grid search

We wanted to test a second method that is robust to local minima in the likelihood landscape. The reason for this is that iterative optimization procedures can get stuck in local minima and not find the globally optimal parameters. We chose to test a two-step grid search method with additional iterative optimization (cf. Mestdagh et al., 2019). Table 6 gives an overview of the method.

We first construct a coarse parameter grid, using values ranging from 2 to 50 with step size 2 for the chain length parameter, and values from 0 to 15 with step size 1 for the β parameter. This results in a grid of 16 by 25, with (25 x 16 =) 400 unique parameter combinations. In the grid we save the predictions of the BMS under each of the unique parameter combinations. To generate these predictions we simulated 10,000 responses for each parameter combination, resulting in predicted distributions of each inference)

Next, we use the PDA method (Holmes, 2015; Turner & Sederberg, 2014) to compute the likelihood of the data for each of the parameter combinations in the coarse grid, which provides us with the best fitting ‘coarse’ parameters. Since we check each of the possible parameter combinations this method can be seen as a ‘brute force’ method. That the BMS has only 2 free parameters, of which one is an integer with a restricted range, allows for the use of such a method.

After finding the best-fitting parameter combination in the coarse grid, we construct a fine grid around this best fitting point. This fine grid consists of 7 chain lengths, the optimal coarse plus or minus 3, and of 11 β values, the optimal coarse one plus or minus 5 with a step size of 0.2. We end up with a fine grid of (7 x 11 =) 77 parameter combinations centered at the optimal parameters in the coarse grid.

As in the case of the coarse grid, we compute the likelihood for each of the 77 parameter combinations in the fine grid using the PDA method. We pick the parameter combination with the highest likelihood to obtain the best fitting parameters in the fine grid.

As a last step, we optimize the β parameter, constrained between the optimal fine grid parameter plus or minus 1, to obtain a more fine-grained estimate of β. This optimization is done iteratively as in Method 1. This last step is not done for the chain length as it is an integer.

Table 6 Overview of Method 2: the two-step grid search method

	Step	Description
1a	Make coarse grid	Create 25 by 16 parameter grid with BMS predictions under 400 unique chain length and beta parameter combinations.
1b	Fit to coarse grid	Select optimal parameter combination from the coarse grid by maximizing the summed likelihood using the PDA method.
2a	Make fine grid	Create a 7 by 11 fine grid centered on the optimal parameter combination in the coarse grid.
2b	Fit to fine grid	Select optimal parameter combination from the fine grid by maximizing the summed likelihood using the PDA method.
3	Optimize β	Iteratively optimize β parameter, restricted to range plus or minus the optimal fine grid β parameter, using the chain length found in step 2b.

Results Method 1

Table 7 presents the correlation coefficients between fitted and true parameters for each of the four datasets for Method 1. Fig. 10 presents scatterplots of the fitted and true parameters for the datasets with 27 observations per participant.

For each of the four datasets we find poor correlations (below .423), for the chain length parameter, and poor to fair correlations for the β parameter (between .333 and .607). Together these findings indicate that Method 1, a traditional iterative method, does not satisfactorily recover the true parameters that generated the data.

Table 7 Pearson correlations of simulated and fitted parameters Method 1

Causal structure	Nr. of observations	β parameter	Chain length parameter
Common cause and Chain	27	.607	.195
	54	.446	.423
Common effect	27	.526	.072
	54	.333	.368

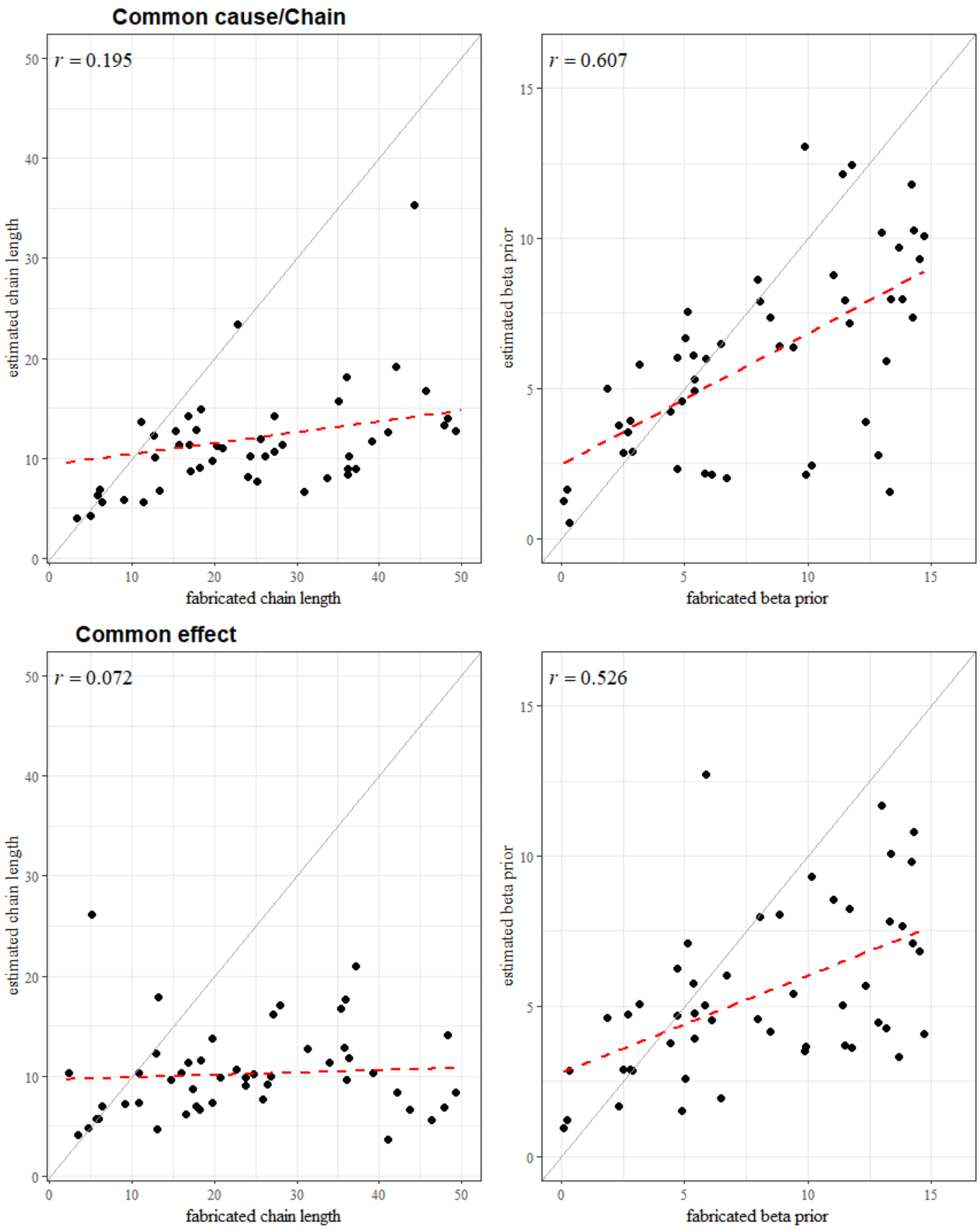

Fig. 10 Scatter plots of true and fitted parameters for Method 1 using 27 observations per participant. Grey diagonal indicates perfect recovery. Dashed red lines indicate the linear trend.

Results Method 2

Table 8 presents the correlation coefficients between fitted and true parameters for each of the four datasets and for each of the steps of Method 2. Note that the *Optimization* column for the chain length parameter in Table 8 is intentionally left blank as the estimate of the chain length parameter does not change in this step.

From Table 8 we can see that Method 2 does accurately recover the true parameters, with all correlations being either good or excellent (between .763 and .949). Notably it is already in the first step of the method, i.e. in the coarse grid, that the correlations are high and that the subsequent steps provide only a marginal improvement in recovery. And this is also the case for the datasets with 27 observations per participant. While the recovery improves consistently with 54 observations, with 27 observations the correlations are already in the range of good to excellent. These findings indicate that one can fit the BMS accurately to data using 27 observations and only a single (coarse) grid.

Fig. 11 presents scatterplots of the true and fitted parameters in each step for the datasets with 27 observations per participant, again the optimization step is left blank for the chain length parameter. From Fig. 11 one can see a notable pattern of lower chain lengths being consistently more accurately estimated than higher chain lengths.

Table 8 Pearson correlations of simulated and fitted parameters Method 2

Causal structure	Nr. of observations	β parameter			Chain length parameter		
		Coarse grid	Fine grid	Optimization	Coarse grid	Fine grid	Optimization
Common cause and Chain	27	. 893	. 895	. 897	.837	.853	-
	54	.926	.930	.938	.918	.928	-
Common effect	27	.897	. 883	. 871	.763	.781	-
	54	.949	.926	.934	.890	.894	-

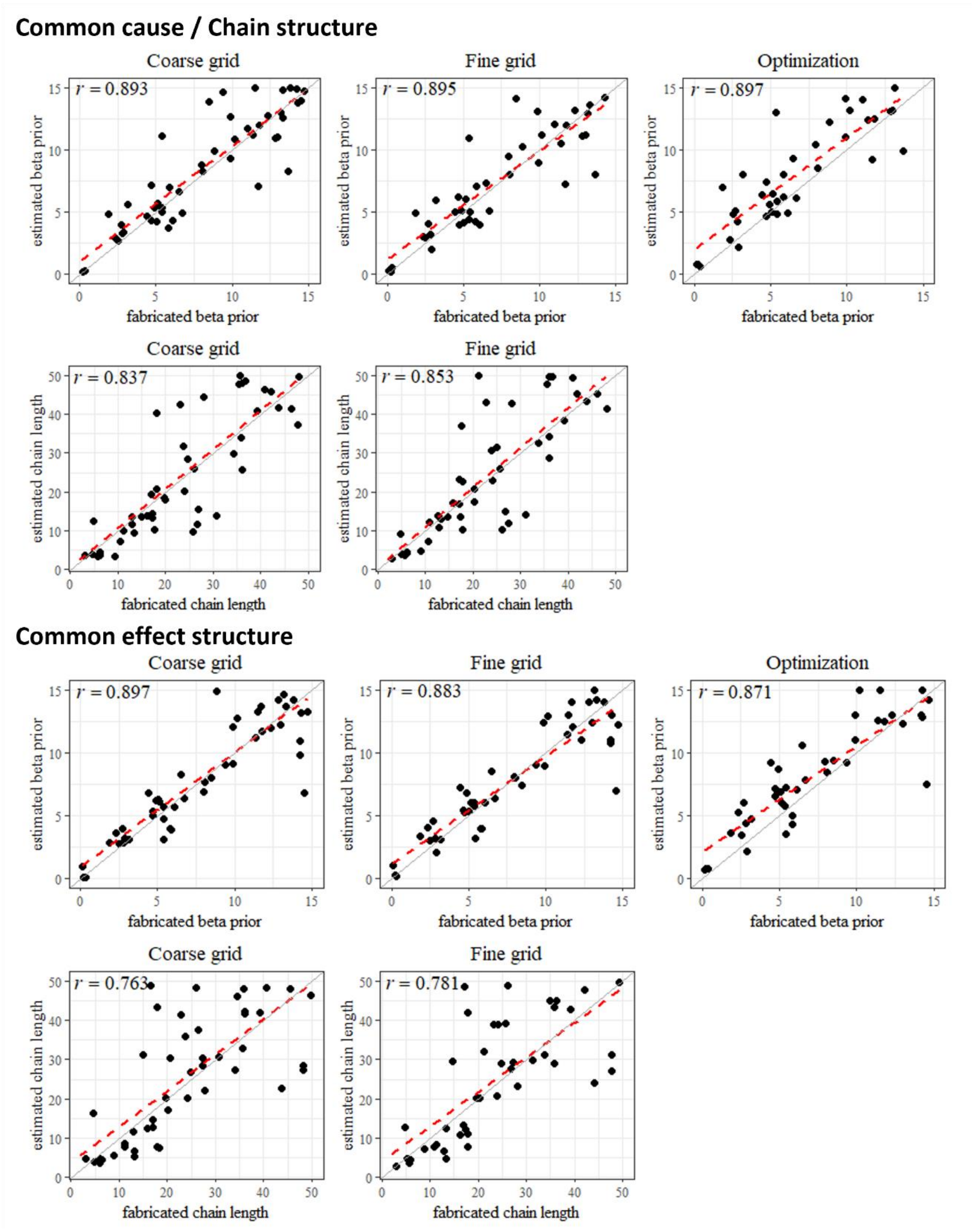

Fig. 11 Scatter plots of true and fitted parameters for Method 2 using 27 observations per participant. Grey diagonal indicates perfect recovery. Dashed red lines indicate the linear trend.

Appendix B: Inferences per inference group

Here we provide a list of inferences per inference group and their normative probability. The causal networks in the experiment were highly symmetric, allowing us to collapse over the terminal variables (e.g. $P(Y = 1|X_1 = 1, X_2 = 0) = P(Y = 1|X_1 = 0, X_2 = 1)$), over the presence or absence of variables (e.g. $P(Y = 1|X_1 = 1, X_2 = 1) = 1 - P(Y = 1| X_1 = 0, X_2 = 0)$), and over unknown variables (e.g. $P(X_1 = 1| Y = 1) = P(Y = 1| X_2 = 1)$). Responses to inferences with an asterisk below (*) are flipped around the midpoint to the upper portion of the probability scale based on the symmetry between the absence and presence of variables (e.g. 25% was converted to 75%; see Rottman & Hastie, 2016; Davis & Rehder 2020). Within each group all inferences have the same normative answer (after flipping) and the BMS predicts the same distribution (after flipping) for each inference in a group.

Conflict trials 1

Inferences with conflicting conditioning information where a terminal variable is queried.

Normative probability: 75%

$P(X_1 = 1|Y = 1,\ X_2 = 0)$

$P(X_2 = 1|Y = 1,\ X_1 = 0)$

$P(X_1 = 1|Y = 0,\ X_2 = 1)$*

$P(X_1 = 1|Y = 0,\ X_2 = 1)$*

Conflict trials 2

Inferences with conflicting conditioning information where the middle variable is queried.

Normative probability: 50%

$P(Y = 1|X_1 = 1,\ X_2 = 0)$

$P(Y = 1|X_1 = 0,\ X_2 = 1)$

Ambiguous trials 1

Inferences where the status is of only one variable is known and this variable is adjacent to the queried variable.

Normative probability: 75%

$P(X_1 = 1|Y = 1)$

$P(X_2 = 1|Y = 1)$

$P(X_1 = 1|Y = 0)$*

$P(X_2 = 1|Y = 0)$*

$P(Y = 1|X_1 = 1)$

$P(Y = 1|X_2 = 1)$

$P(Y = 1|X_1 = 0)$*

$P(Y = 1|X_2 = 0)$*

Ambiguous trials 2

Inferences where the status is of only one variable is known and this variable is not adjacent to the queried variable.

Normative probability: 62.5%

$P(X_1 = 1|X_2 = 1)$

$P(X_2 = 1|X_1 = 1)$

$P(X_1 = 1|X_2 = 0)$*

$P(X_2 = 1|X_1 = 0)$*

Consistent trials 1

Inferences with consistent conditioning information where a terminal variable is queried.

Normative probability: 75%

$P(X_1 = 1|Y = 1,\ X_2 = 1)$

$P(X_2 = 1|Y = 1,\ X_1 = 1)$

$P(X_1 = 1|Y = 0,\ X_2 = 0)$*

$P(X_2 = 1|Y = 0,\ X_1 = 0)$*

Consistent trials 2

Inferences with consistent conditioning information where the middle variable is queried.

Normative probability: 90%

$P(Y = 1|X_1 = 1, X_1 = 1)$

$P(Y = 1|X_1 = 0, X_1 = 0)$*

Base rates

Inferences where no conditioning information is provided.

Normative probability: 50%

$P(X_1 = 1)$

$P(X_2 = 1)$

$P(Y = 1)$

Appendix C: Fit of Mutation Sampler with scaling parameter

The MS was originally fitted using a free 'scaling' parameter *s* such that a predicted response = *s***p*, where *p* is the predicted probability by the MS (Davis & Rehder, 2020). In the main text we have fitted the MS without such a scaling parameter as the scaling parameter can result in part of the predicted distributions falling outside of the response scale. When *s* > 100 (in the original paper it is allowed to vary between 0 and 300), responses above 100% could be produced. For instance, when *s* = 150, and the probability produced by the MS is .90, the MS with scaling would predict a response at 150 x .90 = 135% as we used 0-100% response scale. One could truncate the resultant predicted distribution (i.e. remove the responses above 100% from the prediction), but there is no psychological justification to do so (nor do the original authors do this). As such, the MS with this scaling parameter cannot provide a proper account of the variability of responses as it predicts responses to be outside the response scale.

However, it would behoove us to show that the BMS outperforms the 'published' version of the MS, which includes a scaling parameter[1]. To this end we fitted the MS with a scaling parameter to the data and compared its performance with the BMS.

To fit the MS with a scaling parameter we used the same procedure as in the main text. Davis and Rehder (2020) report scaling parameter estimates ranging from 98 to 130. Based on this we chose a range for *s* symmetric around s = 100 (a value of 100 is equivalent to using no scaling parameter) from 70 to 130. We picked 21 values (as we did for the β parameter) equally spaced in this range, leading to the following set of values for s on the grid: [70, 73, 76, 79, 82, 85, 88, 91, 94, 97, 100, 103, 106, 109, 112, 115, 118, 121, 124, 127, 130].

We find that for 78.0% of the fits the BMS has a lower BIC than the MS with scaling (mean Δ_{BIC} = -14.2). We computed BIC weights as approximations for posterior model probabilities for each participant (Fig. 12). For 38 out of 43 participants (88.4%) the BMS has a higher posterior probability than the MS with scaling factor. Together, these results indicate that the BMS outperforms the MS with scaling.

[1] We thank an anonymous reviewer for pointing this out.

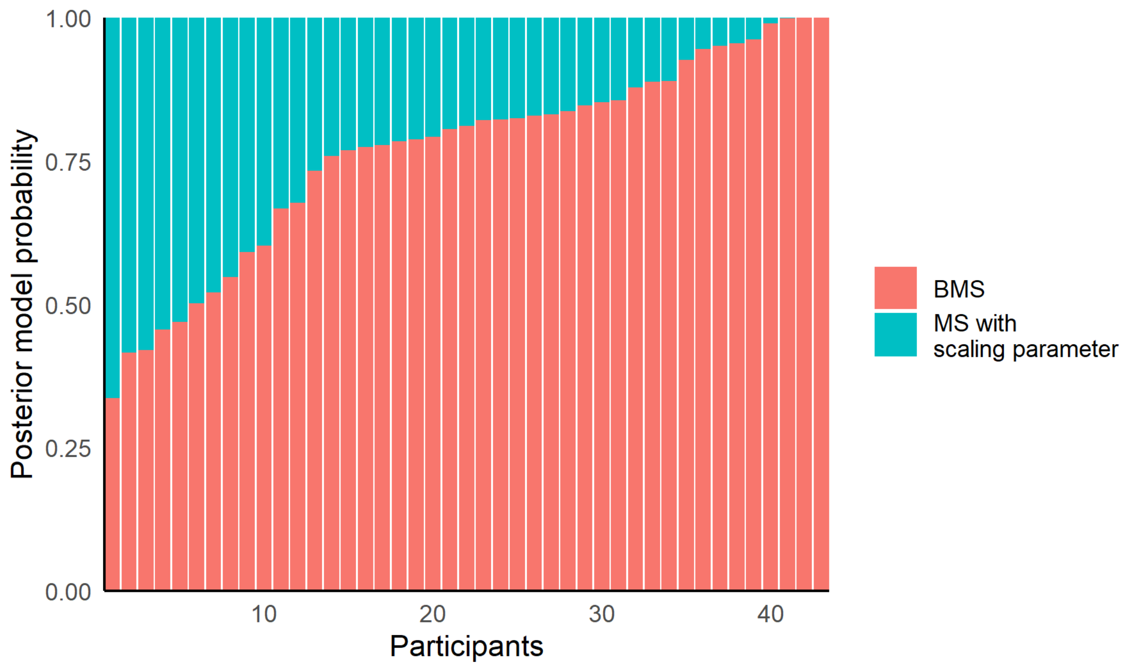

Fig. 12 Posterior model probabilities per participant comparing the BMS and the MS with scaling parameter. Posterior model probabilities are approximated using BIC weights.

Lastly, we find that the mean fitted scaling factor is larger than 100 (*M* = 104.9, *SD* = 14.2). This indicates that indeed the model predicts responses above 100%. Regarding the five participants for whom the MS with scaling factor fit better than the BMS, we find that four of them have an average scaling factor larger than 100 (values: 113.7, 103.7, 113.0, 105.3, 88.0). For these four participants the MS with scaling factor fits better but the model predicts responses above 100%. To illustrate this, Fig. 12 plots the predicted distribution for the inference $P(X_2 = 1|Y = 1,\ X_1 = 0)$ (Conflict trials 1) for one of the participants for whom the MS with scaling was the best fitting model.

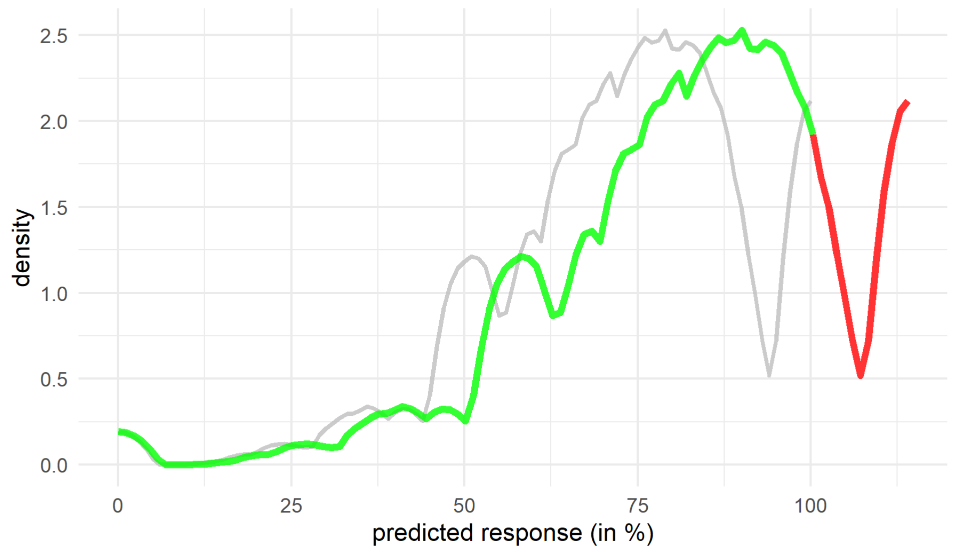

Fig. 13 Predicted distribution of MS with scaling factor. Colored line represents predicted distribution of responses of MS with scaling factor model for inference $P(X_2 = 1|Y = 1,\ X_1 = 0)$ using parameters: chain length = 64, *s* = 113. These parameters are the best fitting parameters for a participant for whom the MS with scaling factor was the best fitting model. The green part of the line indicates part of the distribution that falls within the response scale (0-100%), the red part falls outside the response scale. The thin gray line represents the predicted distribution using the same chain length but without scaling.
